# Landscape of Loci and Candidate Genes for Muscle Fatty Acid Composition in Pigs Revealed by Multiple Population Association Analysis

**DOI:** 10.3389/fgene.2019.01067

**Published:** 2019-10-25

**Authors:** Junjie Zhang, Yifeng Zhang, Huanfa Gong, Leilei Cui, Junwu Ma, Congying Chen, Huashui Ai, Shijun Xiao, Lusheng Huang, Bin Yang

**Affiliations:** State Key Laboratory for Pig Genetic Improvement and Production Technology, Jiangxi Agricultural University, Nanchang, China

**Keywords:** genome-wide association studies, sequence imputation, meta-analysis, fatty acid composition, pig

## Abstract

Genome wide association analyses in diverse populations can identify complex trait loci that are specifically present in one population or shared across multiple populations, which help to better understand the genetic architecture of complex traits in a broader genetic context. In this study, we conducted genome-wide association studies and meta-analysis for 38 fatty acid composition traits with 12–19 million imputed genome sequence SNPs in 2446 pigs from six populations, encompassing White Duroc × Erhualian F_2_, Sutai, Duroc-Landrace-Yorkshire (DLY) three-way cross, Laiwu, Erhualian, and Bamaxiang pigs that were originally genotyped with 60 K or 1.4 million single nucleotide polymorphism (SNP) chips. The analyses uncovered 285 lead SNPs (*P* < 5 × 10^-8^), among which 78 locate more than 1 Mb to the lead chip SNPs were considered as novel, largely augmented the landscape of loci for porcine muscle fatty acid composition. Meta-analysis enhanced the association significance at loci near *FADS2, ABCD2, ELOVL5, ELOVL6, ELOVL7, SCD*, and *THRSP* genes, suggesting possible existence of population shared mutations underlying these loci. Further haplotype analysis at *SCD* loci identified a shared 3.7 kb haplotype in F_2_, Sutai and DLY pigs showing consistent effects of decreasing C18:0 contents in the three populations. In contrast, at *FASN* loci, we found an Erhualian specific haplotype explaining the population specific association signals in Erhualian pigs. This study refines our understanding on landscape of loci and candidate genes for fatty acid composition traits of pigs.

## Introduction

Fatty acid composition affects nutritional value and palatability of meat (Wood et al., 2004). The predominant saturated fatty acids (SFA) in pork are C14:0, C16:0 and C18:0, which together account for about 38% of total fatty acids. From nutritional quality, replacement of the dietary saturated fatty acids (SFA) with monounsaturated fatty acids (MUFA) and polyunsaturated fatty acids (PUFA) are beneficial for human health by lowering low-density lipoprotein cholesterol (FAO, 2010; Sacks et al., 2017), which are risk factors for cardiovascular disease (Katan et al., 1994). Moreover, in term of palatability of meat, the saturated fatty acids are associated with firmness of meat fat, while content of monounsaturated fatty acids, mainly C18:1n-9 and C16:1n-7 is positively correlated with overall acceptability of meat (Cameron et al., 2000). Therefore, a reasonable goal to improve fat quality of pork is to reduce the content of C14:0 and C16:0, and simultaneously increase the percentage of monounsaturated fatty acids, which would produce pork with better nutritional and palatable quality.

Genome-wide association studies (GWAS) based on SNP arrays have successfully identified numerous genomic regions associated with fatty acid composition traits in different pig populations (Ramayo-Caldas et al., 2012; Yang et al., 2013; Ros-Freixedes et al., 2016; Zhang et al., 2016; Zhang et al., 2017). Due to the low density of porcine SNP chips and long range linkage disequilibrium among markers, most of the identified loci have large confidence intervals, which require further fine mapping studies to pinpoint the causal variants. Increasing the marker density and combined analyses on multiple populations can potentially provide a much higher mapping resolution for the trait associated loci (Pausch et al., 2017; Bouwman et al., 2018). Sequencing thousands of individuals can be expensive, a cost-effective approach of increasing marker density is through genotype imputation from a reference population with whole-genome sequences (Druet et al., 2014).

In this study, by referring genome sequences from a panel of 396 individuals, we imputed 34 million SNPs to 2446 pigs from six pig populations that were previously genotyped with 60 K or 1.4 million SNP chips, and conducted genome-wide association and meta-analysis on 38 fatty acid composition traits. The analyses helped to identify new loci and enhanced the association significance of loci detected by chip SNPs. We further investigated the population shared and specific association signatures at *SCD* and *FASN* gene loci, respectively, through haplotype effect and phylogenetic tree analyses. Finally, we performed functional annotation of the lead SNPs with Ensembl tool Variant Effect Predictor (VEP) (McLaren et al., 2010), and based on ChIP-seq peak data of trimethylation at lysine 4 of histone 3 (H3K4me3) and acetylation at lysine 27 of histone 3 (H3K27ac) from 3 porcine liver samples (Villar et al., 2015). In addition, we investigated functional protein–protein interactions (PPI) encoded by candidate genes using the STRING Genomics 10.5 database of PPI network (Szklarczyk et al., 2017). These analyses provided useful information on the genetic architecture and biological pathway underlying the variation in muscle fatty acid composition in pigs.

## Materials and Methods

### Ethics Statements

All the experiments that involved animals were carried out in accordance with the approved guidelines by the Ministry of Agriculture and Rural Affairs of China. The ethics committee of Jiangxi Agricultural University approved the animal experiments in this study.

### Animals and Phenotypes

In this study, six pig populations including the White Duroc × Erhualian F_2_, Sutai, Duroc-Landrace-Yorkshire (DLY) three-way cross, Laiwu, Erhualian, and Bamaxiang pigs were investigated. The detailed information on the six populations has been described (Yang et al., 2013; Liu et al., 2015; Xiong et al., 2015; Zhang et al., 2016; Zhang et al., 2017). Briefly, the F_2_ cross was generated by mating two white Duroc boars and 17 Erhualian sows to produce F_1_ animals, nine F_1_ boars, and 59 F_1_ sows were intercrossed to produce 976 F_2_ males and 945 F_2_ females in six batches. The Sutai pig is a Chinese synthetic breed that is generated by crossing Duroc boars and Taihu sows, and has been selected for prolificacy and growth for more than 18 generations. The Erhualian, Laiwu and Bamaxiang pigs are Chinese indigenous breeds. Erhualian is well known for its high prolificacy. Laiwu is famous for its high intramuscular fat content (average 9∼12%). Bamaxiang has features of two end black coat color and small body size. A total of 385 Erhualian and 390 Laiwu pigs at ages of about 90 days were purchased from Changzhou city in Jiangsu Province and Laiwu city in Shandong Province, respectively. Bamaxiang pigs were purchased from Bama County in Guangxi Province at ages of about 60 days. A total of 698 DLY pigs at ages of 180 ± 3 days were bought from a commercial pig farm from Xiushui city in Jiangxi Province. DLY boars were castrated at ages of about 25 days, and all DLY pigs were fed a corn-soybean diet containing 16% crude protein, 3132 digestible energy and 0.85% lysine. In the F_2_, Sutai, Erhualian, Laiwu and Bamaxiang populations, all piglets were weaned at day 46, males were castrated at day 90. All fattening pigs were raised in consistent indoor condition and fed with corn-soybean based diet containing 16% crude protein, 3100 kJ digestible energy and 0.78 % lysine under standard management. F_2_ and Sutai pigs at 240 ± 5 days, DLY pigs at 180 ± 5 days, and Erhualian, Laiwu and Bamaxiang pigs at 300 ± 5 days were slaughtered in the commercial abattoir.

Fatty acid compositions in *longissimus dorsi* muscle were measured in 591 F_2_, 296 Sutai, 608 DLY, 305 Laiwu, 331 Erhualian and 315 Bamaxiang pigs according to methods as described previously (Folch et al., 1957). We also calculated the ratios of different pairs of fatty acids that reflect the activity of corresponding fatty acids elongase and desaturase using the equations described before (Ulbricht and Southgate, 1991; Pamplona et al., 1998). A total of 38 fatty acid composition traits were obtained for subsequent analysis (**Supplementary Table 1**).

### Genotypes

Genomic DNA of the animals was extracted from ear biopsies using a classic phenol/chloroform method. A total of 1020 F_2_ and 524 Sutai individuals were genotyped with 62,163 SNPs using PorcineSNP60 v1 BeadChip, 610 DLY, 331 Erhualian, and 319 Laiwu pigs were genotyped for 61,565 SNPs using PorcineSNP60 v2 BeadChip (Ramos et al., 2009), and 307 Erhualian and 318 Bamaxiang pigs were genotyped for 1,348,804 SNPs with 1.4 M Affymetrix Axiom SNP chip. In each population, individuals with genotype call rate higher than 90%, SNPs with call rate greater than 90% and the minor allele frequency (MAF) higher than 5% were kept for further analyses. All quality control procedures were performed using PLINK program (Purcell et al., 2007).

A number of 396 sequenced individuals were used as the reference population for genotype imputation. The 396 pigs consist of 221 pigs from 20 Chinese indigenous breeds/populations, 17 Asian wild boars, 140 international commercial pigs, majority of which are Duroc, Large White, Landrace and Pietrain pigs, and 18 European wild boars (**Supplementary Table 2**). Most of the sequencing was conducted by Illumina HiSeq or Xten platforms using 100-150 bp paired-end libraries. The average depth of sequence data is 20.8×, ranged from 4.2× to 45.5×. The sequence data of 232 out of the 396 individuals was generated by our lab, the rest was downloaded from public domain (Groenen et al., 2012; Li et al., 2013; Moon et al., 2015). Among the 396 pigs, two White Duroc and 17 Erhualian pigs are founders of the white Duroc × Erhualian F_2_ population, and 10 Meishan, 12 Bamaxiang, 15 Laiwu, 32 Duroc, 24 Landrace, and 71 Large White have similar or the same ancestry to individuals from six populations investigated in this study.

For most of the sequenced individuals, the raw sequence reads which contains > 50% of base-pairs with base quality score <20 or have >10% of base-pairs are no calls (Ns) were removed. The clean reads of each individual were aligned to *Sus scrofa* reference genome assembly 10.2 using BWA (Li and Durbin, 2009). The mapped reads were sorted using SAMtools (Li et al., 2009), and processed with Indel realignment and duplicate marking in Picard (http://broadinstitute.github.io/picard/). Then, GVCF files were generated from each BAM file using *Haplotypecaller* in GATK by filtering *BadCigar* and *BadMate* reads, and combined using *CombineGVCFs* in GATK. The SNPs were called from combined GVCF files using *GenotypeGVCFs* function in GATK with options of *stand_call_conf 30.0* and *stand_emit_conf 30.0*, and filtered using *VariantFiltration* with option of *QD < 2.0 || FS > 60.0 || MQ < 40.0 || HaplotypeScore > 13.0 || MappingQualityRankSum < -12.5 || ReadPosRankSum < -8.0* according to the best practice guidelines of GATK (McKenna et al., 2010). Finally, a total of 34 million SNPs were called. Haplotypes of the 396 reference animals were constructed using Beagle version 4.0 (Browning and Browning, 2007).

### Genotype Imputation

The 60 K and 1.4 M chip SNPs that have reverse strand compared to genome sequencing SNPs were flipped using PLINK program (Purcell et al., 2007). Then, 34 million SNPs in the 396 sequenced individuals were imputed to the 2446 pigs that were genotyped for 60 K or 1.4 million SNPs using the Beagle v4. After quality control, 14,009,920, 13,686,661, 12,655,741, 13,176,546, 18,282,745 and 18,824,020 SNPs with MAF > 0.05 in 591 F_2_, 296 Sutai, 608 DLY, 305 Laiwu, 331 Erhualian and 315 Bamaxiang pigs, respectively, were retained for subsequent analysis. The accuracy of imputed SNPs were evaluated by Beagle R^2^ values (Browning and Browning, 2007).

### Genome-Wide Association Studies and Meta-Analysis

The association tests were conducted within each population using following model implemented in GEMMA program (Zhou and Stephens, 2012), the same model was also employed to estimate the genomic heritability based on the SNP data:

y=xβ+Zu+e

where **y** is the vector of residual phenotypic values corrected for sex and slaughter batch as fixed effects; **x** is the incidence vector of genotypes of a marker, in which three genotypes AA, AB, and BB of a locus were coded as 0, 1 and 2, respectively, where B is the minor allele. β is the additive allelic effect of the marker; **Z** is identity matrix, **u** is a vector of random polygenic effects that assumed to follow MVN(**0**, **G** $\sigma_{a}^{2}$), where **G** represents the genomic relationship matrix calculated from whole-genome SNP markers and $\sigma_{a}^{2}$ is the additive genetic variance; and **e** is the vector of random residuals, **e** ∼ MVN (0, **I** $\sigma_{e}^{2}$), where **I** is the identity matrix and $\sigma_{e}^{2}$ is the residual variance component. The SNPs that reached a *P* value threshold of 5 × 10^-8^ were empirically considered as significant (Pe'er et al., 2008; Johnson et al., 2010). We defined the most significant SNPs on each chromosome that satisfied *P* value threshold of 5 × 10^-8^ as lead SNPs for a given trait. The phenotypic variation of a trait explained by the lead SNP was calculated by (V_reduce_−V_full_)/V_reduce_, where V_reduce_ and V_full_ are the variance of residuals of ordinary linear model without and with genotypes of the lead SNPs in the models. This estimator was very similar to those calculated using 2p(1-p)a^2^/σ_p_^2^, where p is the allele frequency, and a is additive allelic effect of a given SNP, σ_p_^2^ is phenotypic variance of a trait under study. Meta-analysis of GWAS results on the six populations was performed using a z-score method implemented in METAL software (Willer et al., 2010), which calculated and tested a statistics that combined the effects and standard errors of each SNPs on a given trait in six populations by taking into account sample size and direction of genotype effects in each population. As some of 38 traits investigated in this study are highly correlated to each other, the lead SNPs for different traits can locate closely and in high linkage disequilibrium, we therefore combined the lead SNPs that were within 1 Mb to a same genomic region, and use the lead SNP with strongest association (measured by –log_10_*P* value) as a sentinel SNP for that region (**Table 1**).

**Table 1:** Summary of a selection of sentinel lead SNPs for fatty acid composition and metabolism traits in six pig populations by chromosome regions.

Chromosome: position	Trait	Pop	Candidate genes	*p-*value	MAF (minor/major)	Var (%)^1^	Variant annotation^2^
1:8873447	C18:0	Bamaxiang	*–*	3.02E-08	0.30 (A/G)	11.24	Intron
2:746298	ACL	Erhualian	*–*	2.31E-08	0.07 (G/A)	17.70	Upstream
2:8929954	C20:3n-6/C18:2n-6	Erhualian	*FADS2*, *FADS1*	2.90E-22	0.43 (C/T)	25.05	Intergenic
2:74664653	C20:3n-6	F_2_	*PLIN5*	1.89E-08	0.06 (G/A)	4.66	Intergenic
2:115831690	C20:2n-6	DLY	*–*	5.22E-09	0.06 (C/A)	5.20	Intergenic
2:145804018	FattyAI	Bamaxiang	*–*	5.09E-09	0.38 (T/C)	12.50	Intergenic
3:92010559	C20:1n-9	Erhualian	*–*	9.96E-09	0.33 (T/C)	11.77	Intergenic
4:63910717	C18:1n-9/C18:0	F_2_	*–*	1.69E-10	0.14 (A/G)	9.49	Intron
4:86701762	C20:2n-6	F_2_	*–*	6.22E-09	0.42 (G/T)	9.30	Intergenic
5:73950290	C20:0/C18:0	Sutai	*ABCD2*	2.24E-11	0.25 (A/G)	10.75	Intron
6:67864540	C20:3n-6	Sutai	*–*	2.52E-08	0.27 (T/G)	7.54	Intergenic
6:71008471	C16:1n-7/C16:0	Laiwu	*–*	2.96E-08	0.45 (G/T)	5.61	Intergenic
7:27337970	C18:1n-9/C16:1n-7	Laiwu	*APOM*	4.15E-09	0.35 (C/T)	9.13	Intergenic
7:30614484	C18:1n-9/C16:1n-7	Bamaxiang	*–*	3.40E-09	0.12 (C/T)	11.85	Upstream
7:34919339	n-3	F_2_	*–*	4.93E-15	0.48 (A/G)	15.04	Intron
7:52837555	C20:1n-9	Bamaxiang	*ACSBG1*	8.21E-10	0.24 (G/A)	11.69	Intergenic
7:58228023	C20:2n-6/C18:2n-6	Laiwu	*–*	1.52E-10	0.35 (C/A)	19.97	Intergenic
7:134678195	C20:1n-9/C18:1n-9	Erhualian	*ELOVL5*	1.07E-23	0.22 (G/T)	34.48	Intergenic
8:119726275	C18:1n-9/C16:1n-7	DLY	*ELOVL6*	1.90E-18	0.06 (T/C)	13.06	Intergenic
8:129588470	ACL	DLY	*MTTP*	1.64E-08	0.12 (G/A)	6.35	Intergenic
8:138708016	C20:4n-6/C20:2n-6	Bamaxiang	*SNCA*	3.14E-08	0.06 (A/G)	9.13	Intron
9:11302313	C20:4n-6/C20:3n-6	Erhualian	*DGAT2*	3.13E-08	0.07 (G/A)	7.30	Intron
9:13950534	C16:0/C14:0	Laiwu	*THRSP*	2.91E-08	0.10 (T/C)	17.00	Intron
12:1176481	C18:1n-9/C16:1n-7	Erhualian	*FASN*	2.38E-30	0.07 (T/C)	40.86	Intergenic
12:59707351	SFA	Laiwu	*–*	1.45E-10	0.33 (T/C)	11.23	Intergenic
13:24928872	C20:1n-9/C20:0	Sutai	*ACAA1*	1.14E-09	0.16 (C/T)	15.95	Intron
13:40365857	ACL	Bamaxiang	*ACOX2*	4.00E-09	0.38 (T/G)	11.35	Intergenic
13:165434306	C20:1n-9	Sutai	*–*	3.62E-08	0.07 (A/G)	11.79	Intergenic
14:121454019	C18:0	DLY	*SCD*	3.14E-33	0.33 (T/G)	28.20	Intergenic
15:146109641	C20:0	Laiwu	*–*	3.26E-09	0.42 (C/T)	10.01	Intergenic
16:34617582	C20:1n-9/C20:0	Sutai	*–*	2.45E-08	0.30 (G/A)	16.65	Intergenic
16:36821647	C20:0/C18:0	Laiwu	*–*	1.80E-22	0.09 (T/G)	35.98	Upstream
16:41393886	C20:0/C18:0	F_2_	*ELOVL7*	3.94E-44	0.29 (A/G)	36.26	Intergenic
16:43497948	C20:0/C18:0	DLY	*ELOVL7*	3.66E-62	0.12 (A/T)	43.50	Intergenic
16:48557856	C20:1n-9/C20:0	Bamaxiang	*–*	3.10E-11	0.38 (G/A)	17.33	Intergenic
X:96131734	C14:0	F_2_	*–*	8.49E-09	0.41 (T/A)	6.91	Intergenic
X:103467488	FattyAI	F_2_	*–*	1.63E-10	0.32 (A/C)	8.04	Intergenic

Pop, population; MAF, minor allele frequency. ^1^Var (%), percentage of phenotypic variation explained by the lead SNP for the most significant trait. ^2^Variant annotation, variant annotation by Variant Effect Predictor (VEP) supported by Ensemble; “–”, no candidate gene found.

### Haplotype Analysis

For a given locus, we first extracted the haplotypes of 41 SNPs centered on the lead SNP (20 SNP on each side of the lead SNP), and then estimated the effects of the haplotypes on respective traits using a mixed model similar to that implemented in Nicod et al. (2016) (Nicod et al., 2016):

y=Hβ+Zu+e

Where **H** is a *n*×*p* incidence matrix for the genotype of haplotypes in the populations, *n* is the sample size and *p* is number of haplotypes under study. **β** is a vector of haplotype effects with *p* elements. The **y, Z, u** and **e** are the same as those in the single SNP GWAS model. We multiply both side of the mixed model with inverse matrix of **W**:

$$W^{-1}y=\left(W^{-1}H\right)\beta +W^{-1}\left(\mathrm{Zu}+e\right)$$

Where **W** is the square root of variance and covariance matrix of the phenotypes **V**, which is calculated by $\sigma_{\text{a}}^{2}G+\sigma_{\text{e}}^{2}I$, $\sigma_{\text{a}}^{2}$ and $\sigma_{\text{e}}^{2}$ were estimated in EMMA (Kang et al., 2008). **W** is calculated through eigenvalue decomposition of **V** in R program. After the transformation, the haplotype effects can be estimated by an ordinary linear model implemented in R program. We generated a phylogenetic tree of haplotypes using neighbor joining method implemented in MEGA7 program (Kumar et al., 2016).

### Variants Annotation and Gene Functional Analysis

The genomic position of all SNP used in GWAS were annotated based on *Sus scrofa* 10.2 assembly (Groenen et al., 2012). To facilitate the comparison of our results with those based on *Sus scrofa* 11.1 assembly, we also converted the coordinates of lead SNPs to *Sus scrofa* 11.1 assembly using Liftover (https://genome.ucsc.edu/cgi-bin/hgLiftOver) (**Supplementary Table 3**). All lead SNPs were annotated using the Variant effect predictor (VEP) (Ensembl release 89) (McLaren et al., 2010). We searched for functionally plausible candidate genes in regions within 500 kb of the lead SNPs using UCSC web browser (https://genome.ucsc.edu), which provide homologous genes of different species in the respective QTL region identified in this study. We chose the genes with function relevant to fatty acid or lipid metabolism as candidate genes through literature searching. STRING v10.5 was utilized to examine the candidate genes in context of protein–protein interactions (PPI) network (Szklarczyk et al., 2017). Only interactions with a high level of confidence (score >0.4) were retained in the global PPI network. ClueGO in Cytoscape was employed to implement gene ontology (GO) and the KEGG pathway enrichment analysis (Bindea et al., 2009). The enrichment analysis was carried out using right-sided hypergeometric test, corrected for multiple testing using Benjamini-Hochberg approach. Kappa statistics were used to group the enriched terms (Bindea et al., 2009). The minimum connectivity of the pathway network (kappa score) was set to 0.4.

## Results

### Accuracy of Genotype Imputation

The average Beagle R^2^ values of the SNPs used GWAS are 0.754, 0.756, 0.741, 0.759, 0.892 and 0.893 for F_2_, Sutai, DLY, Laiwu, Erhualian and Bamaxiang pigs, respectively. The Beagle R^2^ is positively associated with minor allele frequency of the SNPs (**Supplementary Figure 1**). Much higher average imputation accuracy was observed in Erhualian and Bamaxiang pigs, because these two populations were genotyped with 1.4 million SNPs, which have much higher marker density compared to the 60 K SNP genotyped for the rest of four populations.

### Genome Wide Association Studies Within Each Population

We estimated genomic heritability (*h_g_*^2^) for the 38 fatty acid composition traits in each of the six populations based on the whole genome SNPs, the average of *h_g_*^2^ estimates for the 38 traits in the six populations is 0.46. A total of 125(54.8%) *h_g_*^2^ estimates were between 0.3 and 0.6, and 48 (21.1%) were greater than 0.6. The *h_g_*^2^ estimated from imputed SNPs were highly correlated with those estimated from chip SNPs (Zhang et al., 2019) (**Supplementary Figure 2**).

We performed genome wide association studies for 38 fatty acid composition traits using the imputed SNPs in each of the six populations. We defined the most significant SNPs on each chromosome that satisfied *P* value threshold of 5 × 10^-8^ as lead SNPs or QTL for a given trait. In total, we identified 131 lead SNPs on 15 porcine chromosomes (**Supplementary Table 3**). The number of lead SNPs for each trait range from 0 to 3, and is positively associated with *h_g_*^2^ (P_spearmancorrelation_ = 2 × 10^-12^) (**Supplementary Figure 3**). A total of 29 lead SNPs that located at more than 1 Mb from the lead SNPs identified by SNP arrays were considered as novel. Additionally, 27 lead SNPs have at least 2 units of increase (range from 2 to 10) in association strength (–log_10_*P* value) compared to those identified based on 60K or 1.4M chips were regarded as enhanced (**Supplementary Table 3**).

**Figure 1:**
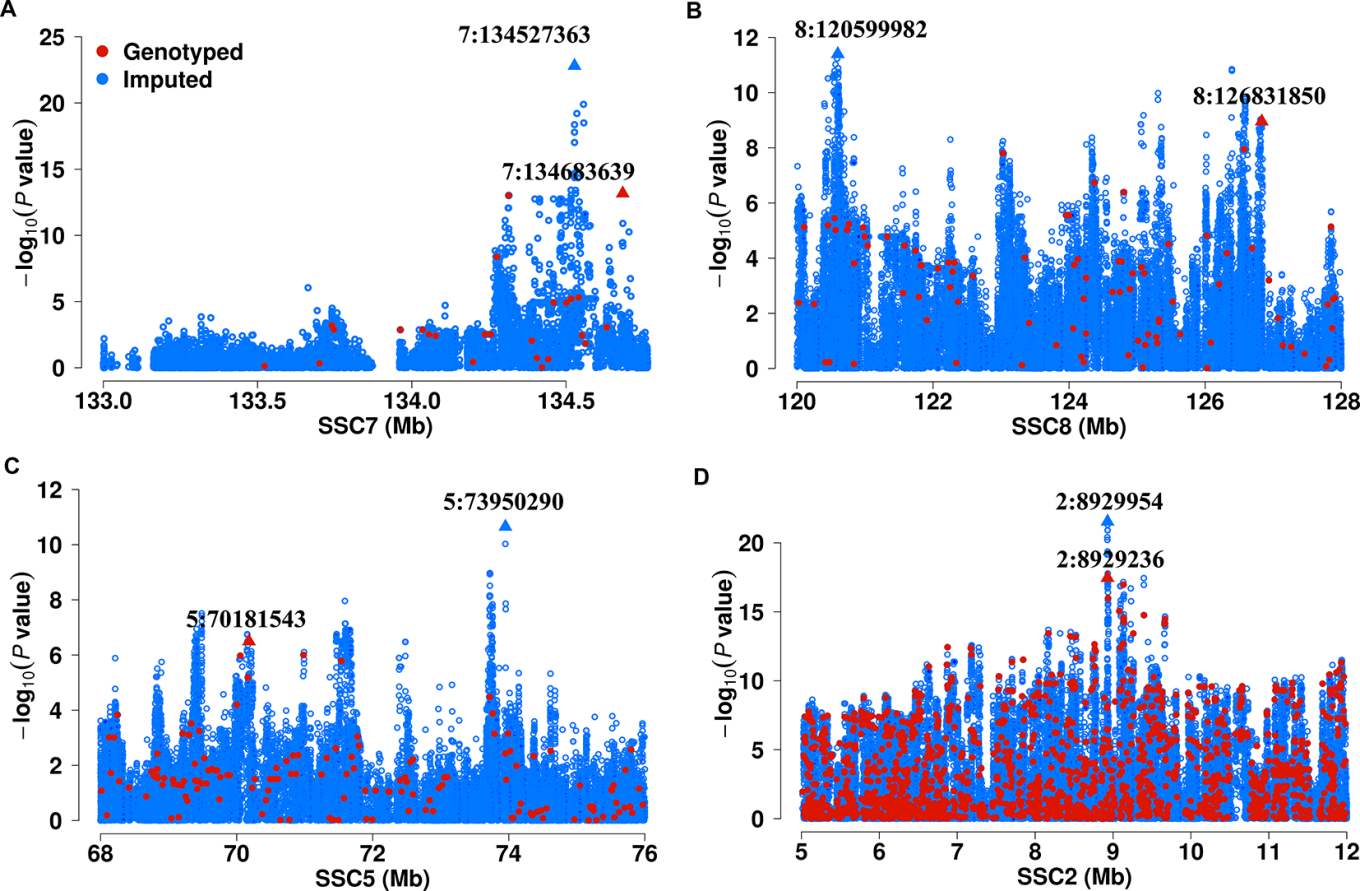
Comparison of association strength between imputed and chip SNPs. **(A)** region for C20:1n-9 on SSC7 in F_2_ population; **(B)** region for C18:1n-9/C16:1n-7 on SSC8 in F_2_ population. **(C)** region for C20:0/C18:0 on SSC5 in Sutai population. **(D)** region for C20:3n-6/C18:2n-6 on SSC2 in Erhualian population. Imputed and chip SNPs were denoted in blue and red color, respectively. The lead SNPs are marked by triangles.

**Figure 2:**
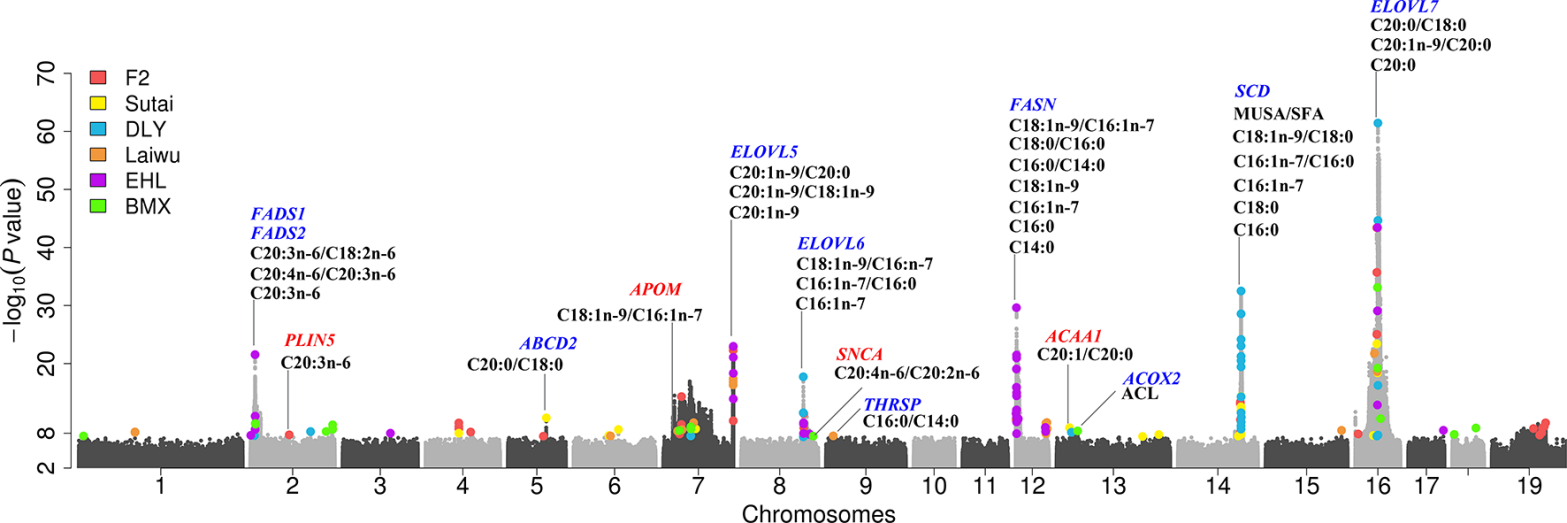
A combined Manhattan plot for GWAS on fatty acid composition traits across six populations. Genome-wide representation of all lead SNPs identified single population GWAS, which were marked by a colored dot. Results from different populations were represented by different colors. The *y* axis shows the -log_10_*p-*values for association with corresponding fatty acid composition traits and the *x* axis shows the genomic position of genetic variants. Candidate genes are denoted with different colors, blue for candidate gene previously identified, and red for candidate gene newly found in current study.

We next show four examples that GWAS based on imputed SNPs improved upon the results obtained from chip SNPs. In the F_2_ population, we previously identified a significant lead SNP (7:134683639, *P* = 6.80 × 10^-14^) for C20:1n-9 on SSC7 that explain 21.0% of phenotypic variance based chip SNPs. By contrast, association of the imputed SNP (7:134527363) achieve a *P* value of 1.58 × 10^-23^ and explain 31.2% of phenotypic variance for C20:1n-9 (**Figure 1A**). Another example is that the top associated variant (8:126831850, *P* = 1.11 × 10^-9^) for C18:1n-9/C16:1n-7 on chromosome 8 found in 60K chips is less significant than, and locate approximately 6 Mb from the top SNP (8:120599982, *P* = 3.99 × 10^-12^) detected hereby (**Figure 1B**). Notably, the newly identified SNP locates closer to *ELOVL6*, the most functionally plausible gene in this region, and those SNPs identified in DLY and Erhualian populations (Zhang et al., 2016; Zhang et al., 2017) (**Supplementary Table 3**). In Sutai pigs, 5:73950290 for C20:0/C18:0 (*P* = 2.24 × 10^-11^) on SSC5 identified here shows greater association significance than the one (5:70181543, 3.20 × 10^-7^) identified based on 60 K SNP data (**Figure 1C**). In Erhualian pigs, the lead SNP (2:8929954, *P* = 2.90 × 10^-22^) for C20:3n-6/C18:2n-6 currently identified was more significant than the loci (2:8929236, *P* = 3.48 × 10^-18^) previously detected using 1.4 M SNP chip (**Figure 1D**). These examples demonstrate GWAS based on the imputed genome sequence SNPs helped to refine the signals revealed by 60K SNP data (**Figure 2**, **Table 1** and **Supplementary Table 3**).

### Meta-Analysis of Genome-Wide Association Studies

**Figure 3:**
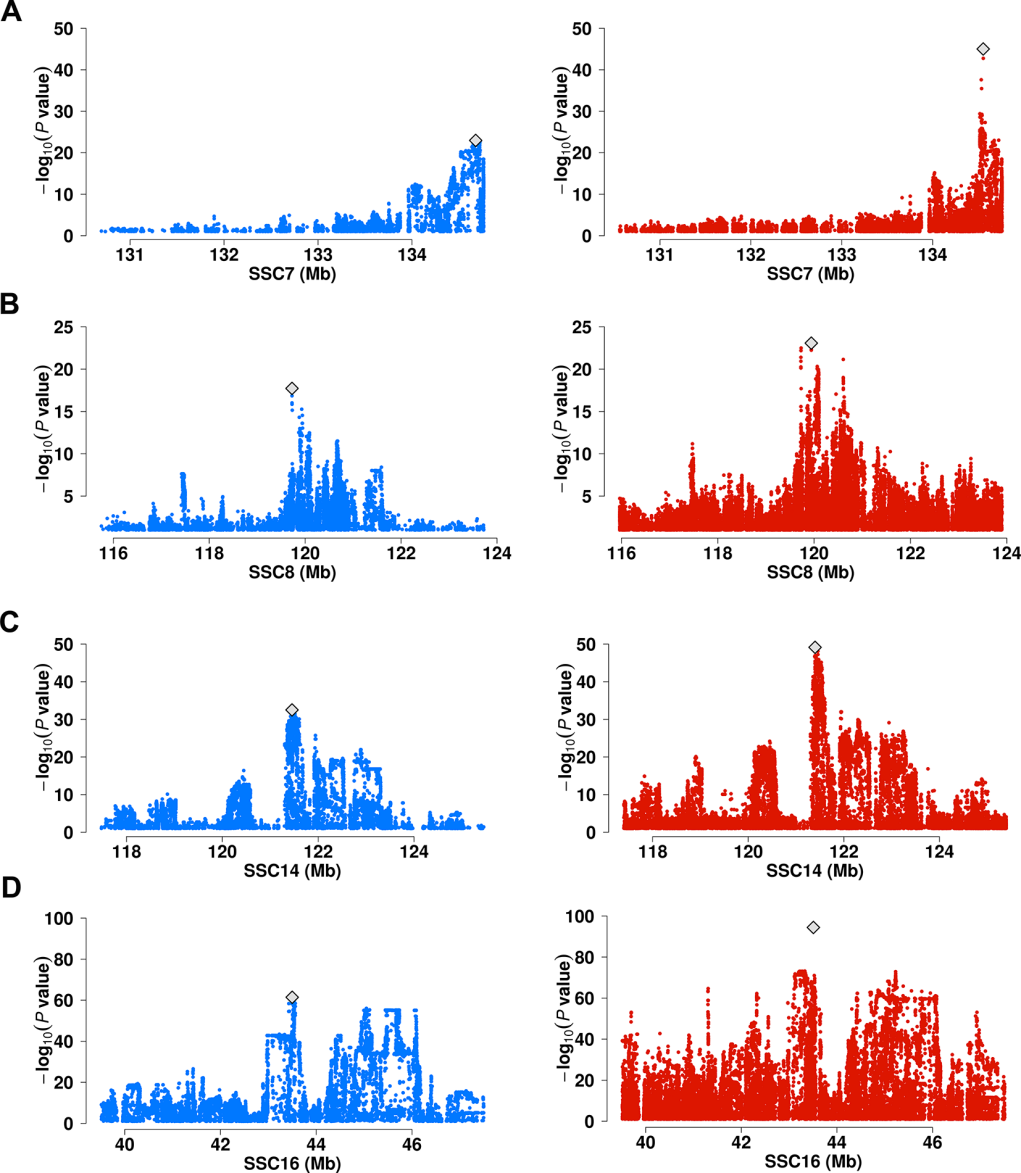
Comparison of association strength between signal-population GWAS and Meta-analysis. **(A)** Associations of SNP in a 4 Mb region on SSC7 for C20:1n-9/C18:1n-9 in Erhualian pigs (blue dots) versus GWAS meta-analysis (red dots). **(B)** Associations of SNP in an 8 Mb region on SSC8 for C18:1n-9/C16:1n-7 in DLY pigs (blue dots) versus GWAS meta-analysis (red dots). **(C)** Associations of SNP in an 8 Mb region on SSC14 for C18:0 in DLY pigs (blue dots) versus GWAS meta-analysis (red dots). **(D)** Associations of SNP in an 8 Mb region on SSC16 for C20:0/C18:0 in DLY pigs (blue dots) versus GWAS meta-analysis (red dots). The lead SNPs were marked with gray diamond.

We performed a meta-analysis on the genome wide association statistics from the six populations. A total of 154 lead SNPs (*P* < 5× 10^-8^) were identified (**Supplementary Table 4**). Among these, 49 locates >1 Mb from the lead SNPs identified in single population GWAS. Moreover, we observed 5 lead SNPs displaying more than 5 units of –log_10_*P* values enhancement upon those of lead SNPs identified in a single population (**Figure 3**, **Supplementary Table 3**, and **Supplementary Table 4**). These include 7:134556509 for C20:1n-9/C18:1n-9 near *ELOVL5* gene (*P* = 9.62 × 10^-46^ vs. *P* = 1.07 × 10^-23^ at 7: 134678195 in Erhualian pigs) (**Figure 3A**), 8:119942096 for C18:1n-9/C16:1n-7 near *ELOVL6* gene (*P* = 8.51 × 10^-24^ vs. *P* = 1.90 × 10^-18^ at 8:119726275 in DLY pigs) (**Figure 3B**), 14:121398370 for C18:0 near *SCD* gene (*P* = 7.34 × 10^-50^ vs. *P* = 3.14 × 10^-33^ at 14:121454019 in DLY pigs) (**Figure 3C**) and 16:43507850 for C20:0/C18:0 near *ELOVL7* gene (*P* = 3.80 × 10^-95^ vs. *P* = 3.66 × 10^-62^ at 16:43497948 in DLY pigs) (**Figure 3D**). The other loci that were enhanced in meta-analyses located near *FADS2, ABCD2*, and *THRSP* genes (**Supplementary Table 4**).

### Haplotype Analyses of Population-Shared and Specific Loci

Next, we carried out haplotype analyses on two QTL regions near *SCD* and *FASN* genes based on following considerations: (1) both regions are strongly associated with multiple primary saturated and mono-unsaturated fatty acids that are closely related to fat quality of meat; (2) both *SCD* and *FASN* genes have direct functional relevance to the fatty acid composition traits. (3) Meta-analysis largely enhanced association significance of *SCD* loci but not *FASN* loci, it is of interest to investigate the effects and phylogeny of haplotypes in these two regions in the six populations to further clarify the phenomenon.

**Figure 4:**
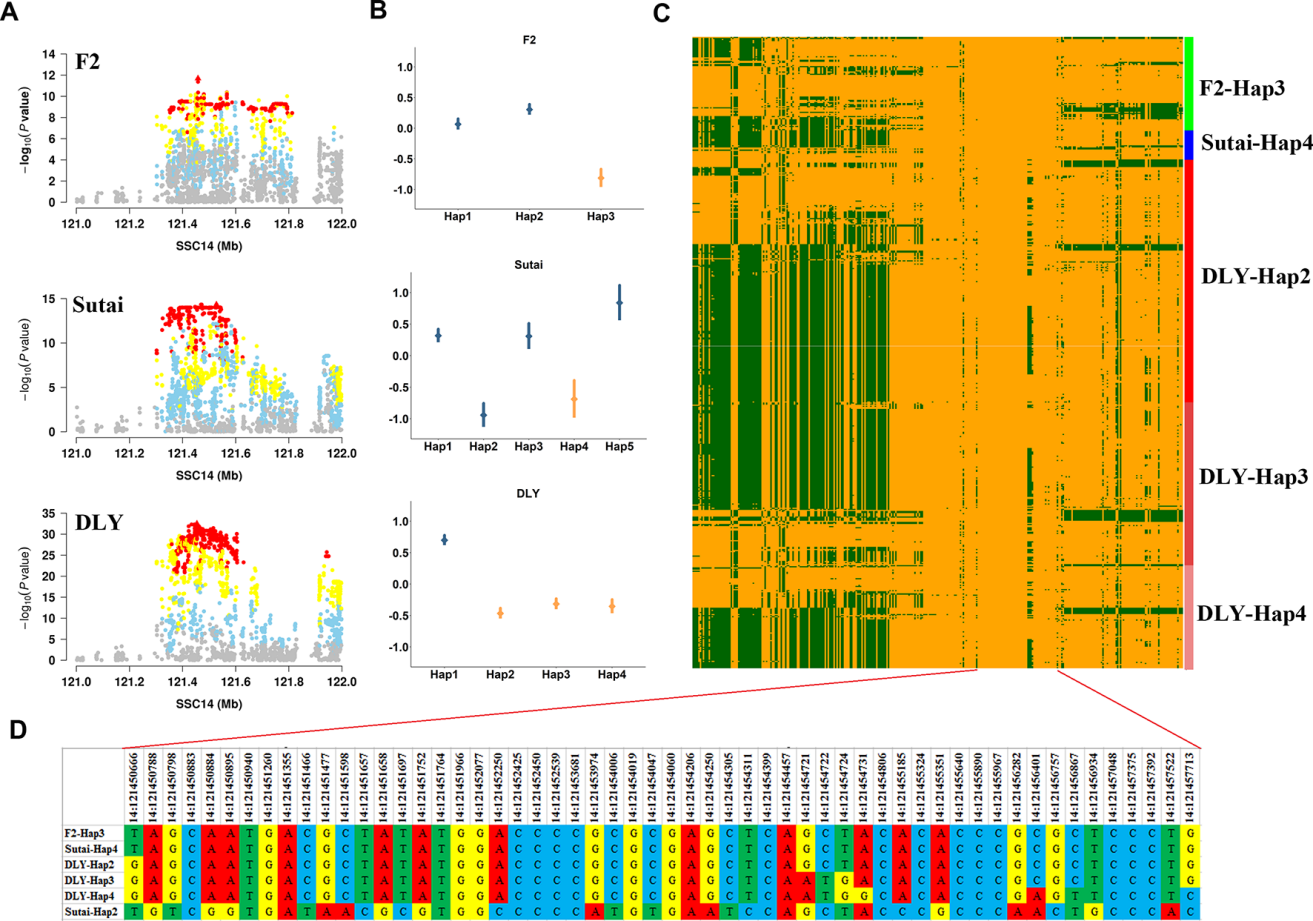
Haplotype analysis of the major QTL for C18:0 on SSC14 in the F_2_, Sutai and DLY populations. **(A)** The significant regional plots for the SNP that affects the C18:0 content on SSC14 across three populations. **(B)** Distribution of effects of 41 SNP haplotype centered on the lead SNP for C18:0 identified in DLY pigs. The points represent estimates of the haplotype effects. Vertical bars represented the standard errors of the haplotype effect estimates. Haplotypes that shared the 3.7 kb segment (121,450,788-121,454,457 bp) were highlighted by orange color. **(C)** Heatmap of haplotypes spans 20kb upstream and downstream (121,434,379-121,473,888 bp) of the lead SNP 14:121,454,019 in individuals that carry Hap3 in F2, Hap4 in Sutai, Hap2, Hap3 and Hap4 in DLY pigs. **(D)** The detail base pair information of a 7-kb segment than encompassed the 3.7 kb (121,450,788-121,454,457 bp) chromosome segment shared by F_2_-Hap3, Sutai-Hap4, DLY-Hap2, DLY-Hap3 and DLY-Hap4 haplotypes.

Near *SCD* gene, we identified lead SNPs within a 1.83 Mb region (between 120.10 and 121.93 Mb) that had significant effects on multiple fatty acid traits (C16:0, C16:1n-7, C18:0 and C18:1n-9) in three populations (F_2_, Sutai, and DLY). Within this region, the most significant association was found between 14:121454019 and C18:0 in DLY pigs (**Figure 4A**). We empirically investigated the effects of 41 SNP haplotypes centered on 14:121454019 in F_2_, Sutai, and DLY pigs (*Materials and Methods*). The **Figure 4B** showed estimated effects of haplotypes with frequency >0.05 in each of the three populations. Notably, we observed that Hap3 in F_2_ pigs (*P* = 1.5×10^-4^), Hap4 in Sutai pigs (*P* = 7.6×10^-8^), and Hap2, Hap3 and Hap4 in DLY pigs (*P* = 2.2×10^-16^) with significant effect of decreasing C18:0 content in three populations share an 3.7-kb segment (121,450,788-121,454,457) (**Figures 4B–D**), these analyses suggested that the coincidence of the association signals in the three populations could be brought about by a same causal mutation located in or linked to the shared 3.7 kb haplotype. One exception is for Hap2 in Sutai pigs, this haplotype also has significant effect of reducing C18:0 content (*P* = 7.6×10^-8^), but do not contain the shared 3.7 kb segment.

**Figure 5:**
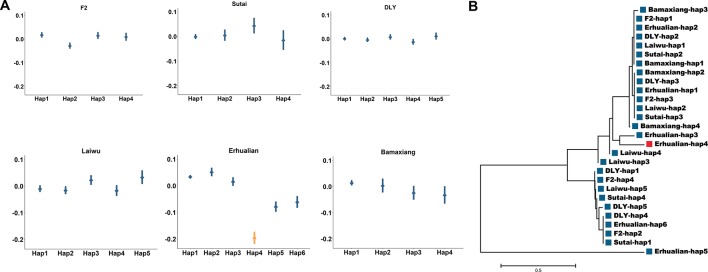
Haplotype analysis of the major QTL for C14:0 on SSC12 in the EHL populations. **(A)** Distribution of effects of 41 SNP haplotype centered on the lead SNP for C14:0 (1,482,028-1,483,956 bp) identified in Erhualian pigs in the six populations. The points represent the estimates of the haplotype effects. Vertical bars represented the standard errors of the haplotype effect estimates. Haplotype (Hap4) with significant effect of decreasing C14:0 in Erhualian pigs was highlighted by orange color. **(B)** Neighbor-joining tree for major haplotypes of the QTL on SSC12 across six populations. Haplotype (Erhualian-Hap4) specific to Erhualian pigs is highlighted by red colors.

Near the *FASN* gene on SSC12, we identified lead SNPs for C14:0 in both Erhualian and Laiwu pigs (**Supplementary Table 3**). The lead SNP (12:1482194, *P* = 3.41 × 10^-22^) identified in Erhualian pigs located at 1.21 Mb from that identified in Laiwu pigs (12:273754, *P* = 4.42 × 10^-11^) (**Supplementary Table 3**). It is of interest to investigate whether a same causative mutation is underlying the association signatures in the two populations. We estimated the effects of haplotypes of 41 SNPs centered on Erhualian lead SNP (12:1482194) in the six populations. Interestingly, we identified a haplotype (Hap4) in Erhualian pigs displaying significant effect (*P* = 4.2 × 10^-19^) on reducing C14:0 content (**Figure 5A**). Phylogenetic analysis on 28 major haplotypes with frequency >0.05 in corresponding population suggested that the Hap4 in Erhualian pigs was not clustered with any haplotypes from the other populations (**Figure 5B**). These analyses suggested that Hap4 that uniquely found in Erhualian pigs underlying the population-specific GWAS signal in Erhualian pigs.

### Annotation and Biological Insights

The lead SNPs identified through GWAS in each of the populations and GWAS meta-analysis correspond to 205 unique lead SNPs. Among these SNPs, 63% locate in intergenic region, 26% locate in introns of genes, 10% located in upstream or downstream regions of genes, and 1% are synonymous variants (**Supplementary Table 3** and **Supplementary Table 4**). Majority of these SNPs are not coding variants. We therefore further annotated these SNPs using published H3K27ac and H3K4me3 peaks (Villar et al., 2015), and found that a total of 15% of the 205 variants locate within the ChIP–seq peaks (H3K27ac: 12.7% and H3K4me3: 2.5%), representing a 2.5 fold enrichment compared with the whole genome SNPs under investigations (H3K27ac: 5.4% and H3K4me3: 0.75%) (**Supplementary Table 3** and **Supplementary Table 4**).

**Figure 6:**
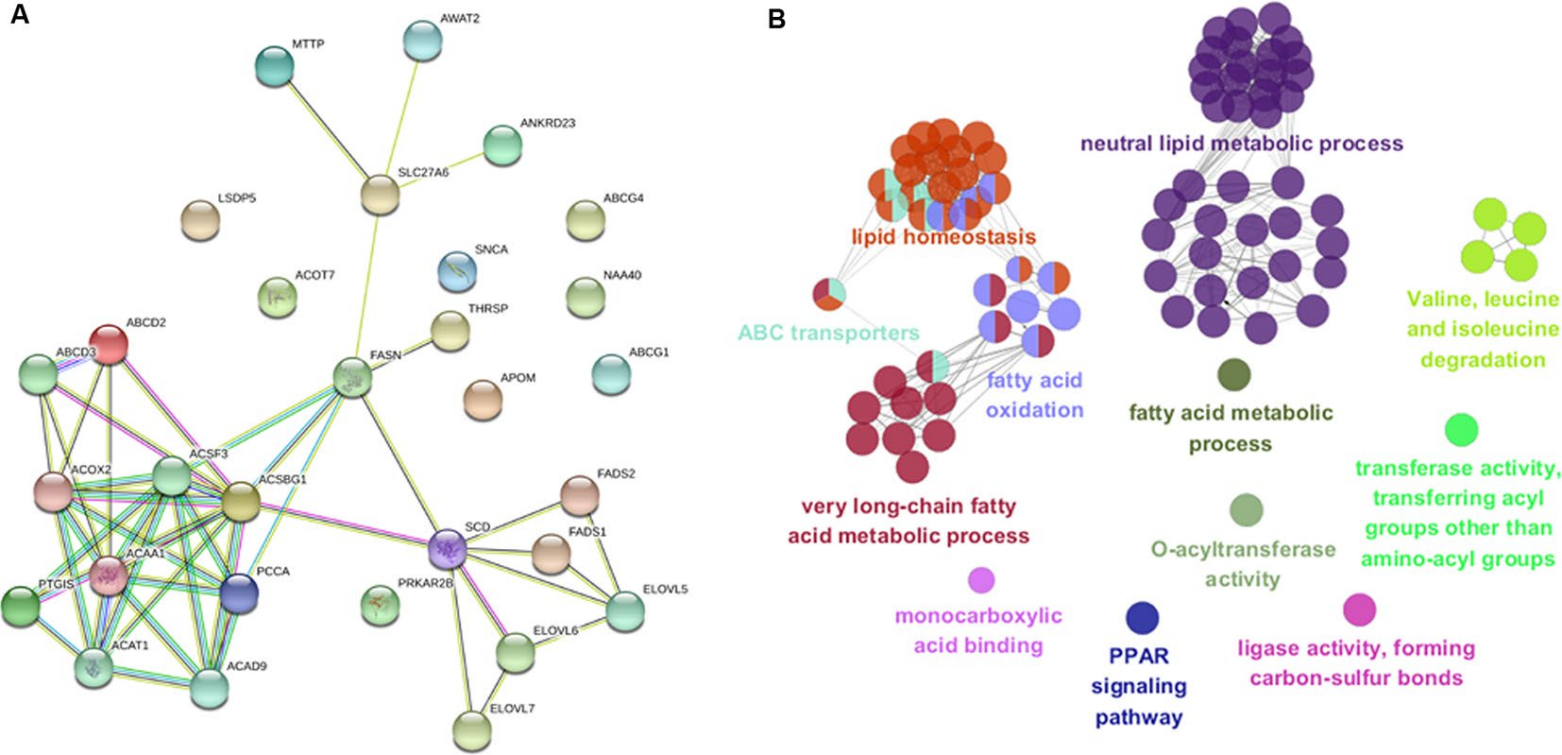
Protein-protein interaction network and gene ontology enrichment analysis of candidate genes. **(A)** Protein-protein interaction network of the 32 most plausible candidate genes of the lead SNPs detected by GWAS and meta-analysis in STRING v10.5 database. **(B)** Gene ontology enrichment analysis of candidate genes. Over-represented GO/pathway terms were grouped based on kappa statistics. GO/pathway terms are represented as nodes, and the node size represents the term enrichment significance, while the edges represent significant similarity between categories.

To gain insight into biological pathways underlying the variations in muscle fatty acid composition in pigs, we investigated the 32 candidate genes that were found within 500 Kb region of the lead SNPs with function relevant to fatty acid or lipid metabolism in context of protein-protein interaction network in STRING database (Szklarczyk et al., 2017). Interestingly, the 32 candidate genes appeared to be highly connected among each other, and several of newly identified candidate genes were evidenced to link to previously identified genes such as *FASN* and *SCD* (**Figure 6A**) (Zhang et al., 2016; Zhang et al., 2017). Gene ontology enrichment analysis highlighted metabolic processes of fatty acids (*P* = 2.2 × 10^-32^), neutral lipid (*P* = 1.6 × 10^-17^) and long-chain fatty acid (7.6 × 10^-17^) as the most enriched terms (**Figure 6B** and **Supplementary Table 5**), this is expected as the 32 genes were chosen according to their functional relevance to fatty acids. Nevertheless, it is still of interest to observe that many genes related to fatty acid catabolism or synthesis located in the vicinity of the fatty acid associated loci, suggesting fatty acid catabolism and synthesis are the primary biological mechanisms that affect the muscle fatty acid composition in pigs.

## Discussion

Identification of genetic variants for fatty acid compositions would provide a cost effective way to improve pork fat quality. Previously, we have performed GWAS for fatty acid composition traits in the six populations based on 60 K or 1.4 M SNP chips (Yang et al., 2013; Zhang et al., 2016; Zhang et al., 2017). Imputation based GWAS had helped to identify missing QTL for lumbar number in Sutai pigs (Yan et al., 2017) and hematological traits in the F_2_ pigs (Yan et al., 2018). In this study, by performing GWAS and meta-analyses based on imputed genome sequence variants, we refined previously identified loci and reveal a number of new loci for fatty acid composition traits. Moreover, the imputed data allowed us investigate the population shared and specific QTL at higher resolution. Especially, integrating the GWAS signals that shared across populations e.g., at SCD loci, helped to greatly refine the QTL.

The genotype accuracy of the imputed SNPs is critical for the success of the association study. Previously, same imputation strategy was employed to reveal solid new loci for vertebral number and hematological traits in the same populations to those investigated in current study (Yan et al., 2017; Yan et al., 2018), which demonstrated that imputing from relatively low density SNP chip data e.g., 60K SNP genotypes, to whole sequence SNPs provide a valuable strategy to improve the GWAS detection precision and power (**Supplementary Figure 1**). A total of 78 out of the 285 lead SNPs detected in this study were considered as new loci. Although most of the newly identified loci showed moderate association significance, with *P* values ranged from 1.10 × 10^-10^ to 4.54 × 10^-8^, we found a number of functional plausible candidate genes near these loci. For instance, in F_2_ pigs, 2:74664653 for C20:3n-6 (*P* = 1.89 × 10^-8^) located 133 Kb from *PLIN5* gene, which plays a crucial role in the regulation of intracellular fatty acid fluxes and oxidation (Laurens et al., 2016). In Sutai pigs 5:73950290 for C20:0/C18:0 (*P* = 2.24 × 10^-11^) on SSC5 located about 200 kb from *ABCD2* (73.68 – 73.74Mb) gene that involved in very long chain fatty acid catabolic process (van Roermund et al., 2011). The lead SNP (13:24928872) for C20:1n-9/C20:0 was approximately 240 Kb from *ACAA1* (Acetyl CoA-Acyl Transferase), this gene encode an enzyme important for β-oxidation of fatty acids in peroxisomes (Zha et al., 2005). The locus for C18:1n-9/C16:1n-7 on SSC7 at 27.3 Mb in Laiwu pigs was adjacent to *APOM* (27.48 Mb), with function related to lipid and lipoprotein metabolism. *APOM* gene was reported to be significantly associated with fat deposition traits in pigs (Pan et al., 2010). In human, it was showed that down-regulation of APOM expression can be induced by palmitic acid (Luo et al., 2014). In Erhualian pigs, the lead SNP at 11.30 Mb for C20:4n-6/C20:3n-6 is near to *DGAT2*, a gene associated with long chain fatty acid metabolism (Cases et al., 2001). In Bamaxiang pigs, the novel lead SNP for C20:4n-6/C20:2n-6 at 138.71 Mb on SSC8 locates in intron of *SNCA* gene. In mice, previous studies demonstrated that ablation of *SNCA* reduces C20:4n-6 turnover and increases C22:6n-6 incorporation in brain phospholipids (Golovko et al., 2006; Golovko et al., 2007).

Moreover, the meta-analyses revealed a number of new candidate genes involved in metabolism/transport of fatty acid or lipid. These included *NAA40* (lipid metabolic process), *SLC27A6* (Transport of long-chain fatty acids), *ANKRD23* (A nuclear protein involved in energy metabolism), *ABCD3* (Peroxisome biogenesis, oxidation of dicarboxylic acids), *ACSF3* (fatty acid biosynthetic process), *ACOT7* (acyl-CoA metabolic process), *ACAT1* (catalyzes the reversible formation of acetoacetyl-CoA), *ABCG4* (cellular cholesterol homeostasis), *PRKAR2B* (regulating energy balance and adiposity), *PCCA* (alpha subunit of the heterodimeric mitochondrial enzyme Propionyl-CoA carboxylase), *ACAD9* (catalyze the rate-limiting step in the beta-oxidation of fatty acyl-CoA), *ABCG1* (phospholipids transport), *PTGIS* (monooxygenases which catalyze and synthesis of cholesterol, steroids and other lipids), *AWAT1-AWAT2* (diacylglycerol acyltransferase) (**Supplementary Table 4**).

Furthermore, in additional to functionally plausible candidate genes, we also found several new lead SNPs overlapping with those loci identified in other populations. For instance, the lead SNP for C14:0 at 134.70 Mb on SSC4 is close to the region (136.10–136.33 Mb on SSC4) for C16:1n-7 evidenced in Iberian × Landrace F_2_ cross (Munoz et al., 2013). The lead SNP for C20:3n-6 at 135.72 Mb on SSC6 near *LEPR* gene is close to the loci for *longissmus* muscle SFA, PUFA, and PUFA/SFA contents in Duroc pigs (Ros-Freixedes et al., 2016). The lead SNP for C20:1n-9/C20:0 at 24.93 Mb on SSC13 in Sutai pigs coincides with the 24.49 - 25.37 Mb region for back fat C16:0 in the IBMAP population (Munoz et al., 2013). The lead SNP for C20:0 at 146.11 Mb on SSC15 in Laiwu pigs was close to loci at 145.53 Mb for C17:0 in a Duroc pig multigenerational population (van Son et al., 2017). The loci at 148.47 Mb on SSC9 and 57.71 Mb on SSC10 identified in current study are overlapped with 146 – 148 Mb on SSC9 for PUFA in Duroc pigs (Ros-Freixedes et al., 2016) and 55 - 56 Mb for C18:2n-6 and PUFA on SSC10 in Large White pigs (Zappaterra et al., 2018), respectively. Therefore, it is reasonable to believe most of the newly identified loci are not artifacts.

We observed significant associations at *SCD, ELOVL5, ELOVL6, ELOVL7*, and *THRSP* in multiple populations. Therefore, we would have chance to leverage GWAS signals from multiple populations to refine respective QTL at these loci. Meta-analyses in cattle have demonstrate the power of integrating multiple population data to identify a small number of candidate causal variants (Bouwman et al., 2018). Correspondingly, in this study, the meta-analyses largely enhanced the association signals at *SCD*, *ELOVL5, ELOVL6*, and *ELOVL7*, the most significant SNPs identified in meta-analyses at these loci are potential candidates for follow up functional studies.

Through haplotype analyses, we identified a 3.7 kb haplotype (121,450,788-121,454,457) shared across F_2_, Sutai and DLY showing a consistent effect of decreasing C18:0 content. Based on a 660 K SNP chip data, an independent study identified a top SNP for back fat C18:0 content at 121,401,766 bp on SSC14 (van Son et al., 2017), which is about 50 kb from the 3.7 kb region identified in our study. Both regions located about 400 kb downstream of *SCD* gene, therefore, we speculate that the underlying mutation could affect the expression of *SCD* through distant regulation mechanism, and hence affect the C18:0 content in *longissimus* muscle. Further study is required to identify the underlying causative mutation.

We did not find any missense mutations among the lead SNPs identified from single population GWAS or meta-analysis, suggesting the variations in the fatty acid composition in pigs is primarily affected by regulatory variants. This is further supported by the enrichment of lead SNPs in the H3K27ac and H3K4me3 peaks that are representatives of active promoters and enhancers (Villar et al., 2015).

Despite that fatty acid composition traits have been investigated for decades, the underlying molecular pathways that influence the fatty acid composition of *longissimus* muscle remains elusive. Functional annotation and enrichment of candidate genes at identified loci, supported that genes pathways related to metabolism rather than to transport of fatty acids would be primary biological process that affect the fatty acid composition in *longissmus* muscle.

## Conclusion

We performed GWAS and GWAS meta-analysis for 38 fatty acid composition traits in 2446 individuals from six different pig populations based on imputed genome sequencing variants. The analyses identified 78 new associations and refined a number of previously identified loci for fatty acid composition traits. This study demonstrated that genotype imputation from sequencing data help to improve power and precision of GWAS. Leveraging data from multiple populations with diverse genetic background hold great promise to fine map QTL that shared across populations. Evidences of candidate genes with function directly related to fatty acid metabolism and transport near respective QTL deepen our understanding of biological mechanism underlying the porcine fatty acid composition traits. The results generated in this study provide beneficial information for pig breeding program to genetically improve fatty acid profile in pork.

## Data Availability Statement

Publicly available data were analyzed for this study. it can be found at the following links: https://www.ncbi.nlm.nih.gov/sra/ERP001813/ https://www.ncbi.nlm.nih.gov/sra/SRA065461/ https://www.ncbi.nlm.nih.gov/sra/SRP047260/ https://www.ncbi.nlm.nih.gov/sra/SRA096093/ https://www.ncbi.nlm.nih.gov/bioproject/PRJNA238851/ https://dataview.ncbi.nlm.nih.gov/object/PRJNA550237?reviewer=muq35cdjpr0nv5ivec9r1civl2 https://doi.org/10.5061/dryad.7kn7r

## Ethics Statement

The ethics committee of Jiangxi Agricultural University approved the animal experiments in this study.

## Author Contributions

LH conceived and designed the experiment, and revised the manuscript. BY supervised the experiment and data analyses, and wrote part of the manuscript. JZ measured the phenotype, analyzed the data and wrote the manuscript. YZ, HG, LC, JM, CC, HA and SX contributed to experimental materials. All authors read and approved the final manuscript.

## Funding

The work was supported by funds from National Natural Science Foundation of China (31572379 and 31230069).

## Conflict of Interest

The authors declare that the research was conducted in the absence of any commercial or financial relationships that could be construed as a potential conflict of interest.

The reviewer JM declared a past co-authorship with one of the authors SX to the handling editor.

## References

[B1] Bindea G. Mlecnik B. Hackl H. Charoentong P. Tosolini M. Kirilovsky A. (2009). ClueGO: a cytoscape plug-in to decipher functionally grouped gene ontology and pathway annotation networks. Bioinformatics 25 (8), 1091–1093. 10.1093/bioinformatics/btp101 19237447 PMC2666812

[B2] Bouwman A. C. Daetwyler H. D. Chamberlain A. J. Ponce C. H. Sargolzaei M. Schenkel F. S. (2018). Meta-analysis of genome-wide association studies for cattle stature identifies common genes that regulate body size in mammals. Nat. Genet. 50 (3), 362–367. 10.1038/s41588-018-0056-5 29459679

[B3] Browning S. R. Browning B. L. (2007). Rapid and accurate haplotype phasing and missing-data inference for whole-genome association studies by use of localized haplotype clustering. Am. J. Hum. Genet. 81 (5), 1084–1097. 10.1086/521987 17924348 PMC2265661

[B4] Cameron N. D. Enser M. Nute G. R. Whittington F. M. Penman J. C. Fisken A. C. (2000). Genotype with nutrition interaction on fatty acid composition of intramuscular fat and the relationship with flavour of pig meat. Meat Sci. 55 (2), 187–195. 10.1016/S0309-1740(99)00142-4 22061084

[B5] Cases S. Stone S. J. Zhou P. Yen E. Tow B. Lardizabal K. D. (2001). Cloning of DGAT2, a second mammalian diacylglycerol acyltransferase, and related family members. J. Biol. Chem. 276 (42), 38870–38876. 10.1074/jbc.M106219200 11481335

[B6] Druet T. Macleod I. M. Hayes B. J. (2014). Toward genomic prediction from whole-genome sequence data: impact of sequencing design on genotype imputation and accuracy of predictions. Heredity (Edinb.) 112 (1), 39–47. 10.1038/hdy.2013.13 23549338 PMC3860159

[B7] FAO (2010). Fats and fatty acids in human nutrition. Report of an expert consultation. FAO Food Nutr. Pap. 91, 1–166. 21812367

[B8] Folch J. Lees M. Sloane Stanley G. H. (1957). A simple method for the isolation and purification of total lipides from animal tissues. J. Biol. Chem. 226 (1), 497–509. 13428781

[B9] Golovko M. Y. Rosenberger T. A. Faergeman N. J. Feddersen S. Cole N. B. Pribill I. (2006). Acyl-CoA synthetase activity links wild-type but not mutant alpha-synuclein to brain arachidonate metabolism. Biochemistry 45 (22), 6956–6966. 10.1021/bi0600289 16734431 PMC2532510

[B10] Golovko M. Y. Rosenberger T. A. Feddersen S. Faergeman N. J. Murphy E. J. (2007). Alpha-synuclein gene ablation increases docosahexaenoic acid incorporation and turnover in brain phospholipids. J. Neurochem. 101 (1), 201–211. 10.1111/j.1471-4159.2006.04357.x 17250657

[B11] Groenen M. A. Archibald A. L. Uenishi H. Tuggle C. K. Takeuchi Y. Rothschild M. F. (2012). Analyses of pig genomes provide insight into porcine demography and evolution. Nature 491 (7424), 393–398. 10.1038/nature11622 23151582 PMC3566564

[B12] Johnson R. C. Nelson G. W. Troyer J. L. Lautenberger J. A. Kessing B. D. Winkler C. A. (2010). Accounting for multiple comparisons in a genome-wide association study (GWAS). BMC Genomics 11, 724. 10.1186/1471-2164-11-724 21176216 PMC3023815

[B13] Kang H. M. Zaitlen N. A. Wade C. M. Kirby A. Heckerman D. Daly M. J. (2008). Efficient control of population structure in model organism association mapping. Genetics 178 (3), 1709–1723. 10.1534/genetics.107.080101 18385116 PMC2278096

[B14] Katan M. B. Zock P. L. Mensink R. P. (1994). Effects of fats and fatty acids on blood lipids in humans: an overview. Am. J. Clin. Nutr. 60 (6 Suppl), 1017s–1022s. 10.1093/ajcn/60.6.1017S 7977143

[B15] Kumar S. Stecher G. Tamura K. (2016). MEGA7: molecular evolutionary genetics analysis version 7.0 for bigger datasets. Mol. Biol. Evol. 33 (7), 1870–1874. 10.1093/molbev/msw054 27004904 PMC8210823

[B16] Laurens C. Bourlier V. Mairal A. Louche K. Badin P. M. Mouisel E. (2016). Perilipin 5 fine-tunes lipid oxidation to metabolic demand and protects against lipotoxicity in skeletal muscle. Sci. Rep. 6, 38310. 10.1038/srep38310 27922115 PMC5138838

[B17] Li H. Durbin R. (2009). Fast and accurate short read alignment with Burrows-Wheeler transform. Bioinformatics 25 (14), 1754–1760. 10.1093/bioinformatics/btp324 19451168 PMC2705234

[B18] Li H. Handsaker B. Wysoker A. Fennell T. Ruan J. Homer N. (2009). The sequence alignment/map format and SAMtools. Bioinformatics 25 (16), 2078–2079. 10.1093/bioinformatics/btp352 19505943 PMC2723002

[B19] Li M. Tian S. Jin L. Zhou G. Li Y. Zhang Y. (2013). Genomic analyses identify distinct patterns of selection in domesticated pigs and Tibetan wild boars. Nat. Genet. 45 (12), 1431–1438. 10.1038/ng.2811 24162736

[B20] Liu X. Xiong X. Yang J. Zhou L. Yang B. Ai H. (2015). Genome-wide association analyses for meat quality traits in Chinese Erhualian pigs and a Western Duroc x (Landrace x Yorkshire) commercial population. Genet Sel. Evol. 47, 44. 10.1186/s12711-015-0120-x 25962760 PMC4427942

[B21] Luo G. Shi Y. Zhang J. Mu Q. Qin L. Zheng L. (2014). Palmitic acid suppresses apolipoprotein M gene expression via the pathway of PPARbeta/delta in HepG2 cells. Biochem. Biophys. Res. Commun. 445 (1), 203–207. 10.1016/j.bbrc.2014.01.170 24508264

[B22] McKenna A. Hanna M. Banks E. Sivachenko A. Cibulskis K. Kernytsky A. (2010). The genome analysis toolkit: a mapReduce framework for analyzing next-generation DNA sequencing data. Genome Res. 20 (9), 1297–1303. 10.1101/gr.107524.110 20644199 PMC2928508

[B23] McLaren W. Pritchard B. Rios D. Chen Y. Flicek P. Cunningham F. (2010). Deriving the consequences of genomic variants with the Ensembl API and SNP Effect Predictor. Bioinformatics 26 (16), 2069–2070. 10.1093/bioinformatics/btq330 20562413 PMC2916720

[B24] Moon S. Kim T. H. Lee K. T. Kwak W. Lee T. Lee S. W. (2015). A genome-wide scan for signatures of directional selection in domesticated pigs. BMC Genomics 16, 130. 10.1186/s12864-015-1330-x 25765548 PMC4349229

[B25] Munoz M. Rodriguez M. C. Alves E. Folch J. M. Ibanez-Escriche N. Silio L. (2013). Genome-wide analysis of porcine backfat and intramuscular fat fatty acid composition using high-density genotyping and expression data. BMC Genomics 14, 845. 10.1186/1471-2164-14-845 24295214 PMC4046688

[B26] Nicod J. Davies R. W. Cai N. Hassett C. Goodstadt L. Cosgrove C. (2016). Genome-wide association of multiple complex traits in outbred mice by ultra-low-coverage sequencing. Nat. Genet. 48 (8), 912–918. 10.1038/ng.3595 27376238 PMC4966644

[B27] Pamplona R. Portero-Otin M. Riba D. Ruiz C. Prat J. Bellmunt M. J. (1998). Mitochondrial membrane peroxidizability index is inversely related to maximum life span in mammals. J. Lipid Res. 39 (10), 1989–1994. 9788245

[B28] Pan G. Fu Y. Zuo B. Ren Z. Xu D. Lei M. (2010). Molecular characterization, expression profile and association analysis with fat deposition traits of the porcine APOM gene. Mol. Biol. Rep. 37 (3), 1363–1371. 10.1007/s11033-009-9518-2 19326253

[B29] Pausch H. Emmerling R. Gredler-Grandl B. Fries R. Daetwyler H. D. Goddard M. E. (2017). Meta-analysis of sequence-based association studies across three cattle breeds reveals 25 QTL for fat and protein percentages in milk at nucleotide resolution. BMC Genomics 18 (1), 853. 10.1186/s12864-017-4263-8 29121857 PMC5680815

[B30] Pe'er I. Yelensky R. Altshuler D. Daly M. J. (2008). Estimation of the multiple testing burden for genomewide association studies of nearly all common variants. Genet. Epidemiol. 32 (4), 381–385. 10.1002/gepi.20303 18348202

[B31] Purcell S. Neale B. Todd-Brown K. Thomas L. Ferreira M. A. Bender D. (2007). PLINK: a tool set for whole-genome association and population-based linkage analyses. Am. J. Hum. Genet. 81 (3), 559–575. 10.1086/519795 17701901 PMC1950838

[B32] Ramayo-Caldas Y. Mercade A. Castello A. Yang B. Rodriguez C. Alves E. (2012). Genome-wide association study for intramuscular fatty acid composition in an Iberian x Landrace cross. J. Anim. Sci. 90 (9), 2883–2893. 10.2527/jas.2011-4900 22785162

[B33] Ramos A. M. Crooijmans R. P. Affara N. A. Amaral A. J. Archibald A. L. Beever J. E. (2009). Design of a high density SNP genotyping assay in the pig using SNPs identified and characterized by next generation sequencing technology. PLoS One 4 (8), e6524. 10.1371/journal.pone.0006524 19654876 PMC2716536

[B34] Ros-Freixedes R. Gol S. Pena R. N. Tor M. Ibanez-Escriche N. Dekkers J. C. (2016). Genome-wide association study singles out scd and lepr as the two main loci influencing intramuscular fat content and fatty acid composition in duroc pigs. PLoS One 11 (3), e0152496. 10.1371/journal.pone.0152496 27023885 PMC4811567

[B35] Sacks F. M. Lichtenstein A. H. Wu J. H. Y. Appel L. J. Creager M. A. Kris-Etherton P. M. (2017). Dietary fats and cardiovascular disease: a presidential advisory from the american heart association. Circulation 136 (3), e1–e23. 10.1161/cir.0000000000000510 28620111

[B36] Szklarczyk D. Morris J. H. Cook H. Kuhn M. Wyder S. Simonovic M. (2017). The STRING database in 2017: quality-controlled protein-protein association networks, made broadly accessible. Nucleic Acids Res. 45 (D1), D362–d368. 10.1093/nar/gkw937 27924014 PMC5210637

[B37] Ulbricht T. L. Southgate D. A. (1991). Coronary heart disease: seven dietary factors. Lancet 338 (8773), 985–992. 10.1016/0140-6736(91)91846-M 1681350

[B38] van Roermund C. W. Visser W. F. Ijlst L. Waterham H. R. Wanders R. J. (2011). Differential substrate specificities of human ABCD1 and ABCD2 in peroxisomal fatty acid beta-oxidation. Biochim. Biophys. Acta 1811 (3), 148–152. 10.1016/j.bbalip.2010.11.010 21145416

[B39] van Son M. Enger E. G. Grove H. Ros-Freixedes R. Kent M. P. Lien S. (2017). Genome-wide association study confirm major QTL for backfat fatty acid composition on SSC14 in Duroc pigs. BMC Genomics 18 (1), 369. 10.1186/s12864-017-3752-0 28494783 PMC5426056

[B40] Villar D. Berthelot C. Aldridge S. Rayner T. F. Lukk M. Pignatelli M. (2015). Enhancer evolution across 20 mammalian species. Cell 160 (3), 554–566. 10.1016/j.cell.2015.01.006 25635462 PMC4313353

[B41] Willer C. J. Li Y. Abecasis G. R. (2010). METAL: fast and efficient meta-analysis of genomewide association scans. Bioinformatics 26 (17), 2190–2191. 10.1093/bioinformatics/btq340 20616382 PMC2922887

[B42] Wood J. D. Richardson R. I. Nute G. R. Fisher A. V. Campo M. M. Kasapidou E. (2004). Effects of fatty acids on meat quality: a review. Meat Sci. 66 (1), 21–32. 10.1016/s0309-1740(03)00022-6 22063928

[B43] Xiong X. Liu X. Zhou L. Yang J. Yang B. Ma H. (2015). Genome-wide association analysis reveals genetic loci and candidate genes for meat quality traits in Chinese Laiwu pigs. Mamm. Genome 26 (3-4), 181–190. 10.1007/s00335-015-9558-y 25678226

[B44] Yan G. Guo T. Xiao S. Zhang F. Xin W. Huang T. (2018). Imputation-based whole-genome sequence association study reveals constant and novel loci for hematological traits in a large-scale swine F2 resource population. Front. Genet. 9, 401. 10.3389/fgene.2018.00401 30405681 PMC6204663

[B45] Yan G. Qiao R. Zhang F. Xin W. Xiao S. Huang T. (2017). Imputation-Based Whole-Genome Sequence Association Study Rediscovered the missing QTL for Lumbar Number in Sutai Pigs. Sci. Rep. 7 (1), 615. 10.1038/s41598-017-00729-0 28377593 PMC5429657

[B46] Yang B. Zhang W. Zhang Z. Fan Y. Xie X. Ai H. (2013). Genome-wide association analyses for fatty acid composition in porcine muscle and abdominal fat tissues. PLoS One 8 (6), e65554. 10.1371/journal.pone.0065554 23762394 PMC3676363

[B47] Zappaterra M. Ros-Freixedes R. Estany J. Davoli R. (2018). Association study highlights the influence of ELOVL fatty acid elongase 6 gene region on backfat fatty acid composition in Large White pig breed. Animal 12 (12), 2443–2452. 10.1017/s1751731118000484 29580300

[B48] Zha S. Ferdinandusse S. Hicks J. L. Denis S. Dunn T. A. Wanders R. J. (2005). Peroxisomal branched chain fatty acid beta-oxidation pathway is upregulated in prostate cancer. Prostate 63 (4), 316–323. 10.1002/pros.20177 15599942

[B49] Zhang J. Zhang Y. Gong H. Cui L. Huang T. Ai H. (2017). Genetic mapping using 1.4M SNP array refined loci for fatty acid composition traits in Chinese Erhualian and Bamaxiang pigs. J. Anim. Breed. Genet. 134 (6), 472–483. 10.1111/jbg.12297 28940847

[B50] Zhang W. Zhang J. Cui L. Ma J. Chen C. Ai H. (2016). Genetic architecture of fatty acid composition in the longissimus dorsi muscle revealed by genome-wide association studies on diverse pig populations. Genet Sel. Evol. 48, 5. 10.1186/s12711-016-0184-2 26796620 PMC4722735

[B51] Zhang Y. Zhang J. Gong H. Cui L. Zhang W. Ma J. (2019). Genetic correlation of fatty acid composition with growth, carcass, fat deposition and meat quality traits based on GWAS data in six pig populations. Meat Sci. 150, 47–55. 10.1016/j.meatsci.2018.12.008 30584983

[B52] Zhou X. Stephens M. (2012). Genome-wide efficient mixed-model analysis for association studies. Nat. Genet. 44 (7), 821–824. 10.1038/ng.2310 22706312 PMC3386377

