# Supplementary material for: Landscape of Loci and Candidate Genes for Muscle Fatty Acid Composition in Pigs Revealed by Multiple Population Association Analysis
Source: Front Genet. 2019 Oct 25;10:1067. doi: 10.3389/fgene.2019.01067 (PMC6824322; doi:10.3389/fgene.2019.01067)
Supplement: Supplementary file 1 [file DataSheet_1.docx]

# *Supplementary Material*

**Landscape of loci and candidate genes for muscle fatty acid composition in pigs revealed by multiple population association analysis**

Junjie Zhang, Yifeng Zhang, Huanfa Gong, Leilei Cui, Junwu Ma, Congying Chen, Huashui Ai, Jun Ren, Shijun Xiao, Lusheng Huang^*^ and Bin Yang^*^

*State Key Laboratory for Pig Genetic Improvement and Production Technology, Jiangxi Agricultural University, Nanchang, China*

***Correspondence:**

Prof. Lusheng Huang

lushenghuang@hotmail.com

Prof. Bin Yang

binyang@live.cn

**1 Supplementary Figures and Tables**

**1.1 Supplementary Figures**


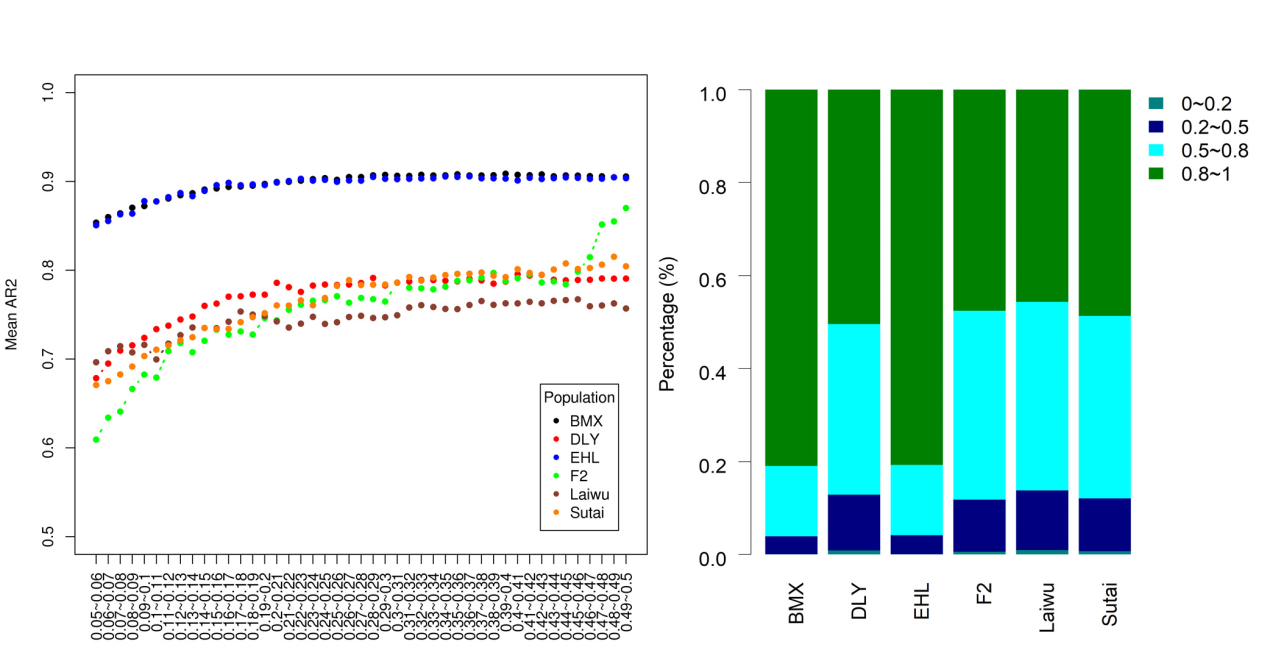


**B**

**A**

**Supplementary Figure S1**. The accuracy of genotype imputation in the six population in terms of Beagle allelic R^2^. **(A)** The relationship between minor allele frequency and imputation accuracy. **(B)** The distribution of Beagle allelic R^2^ values in the six populations.


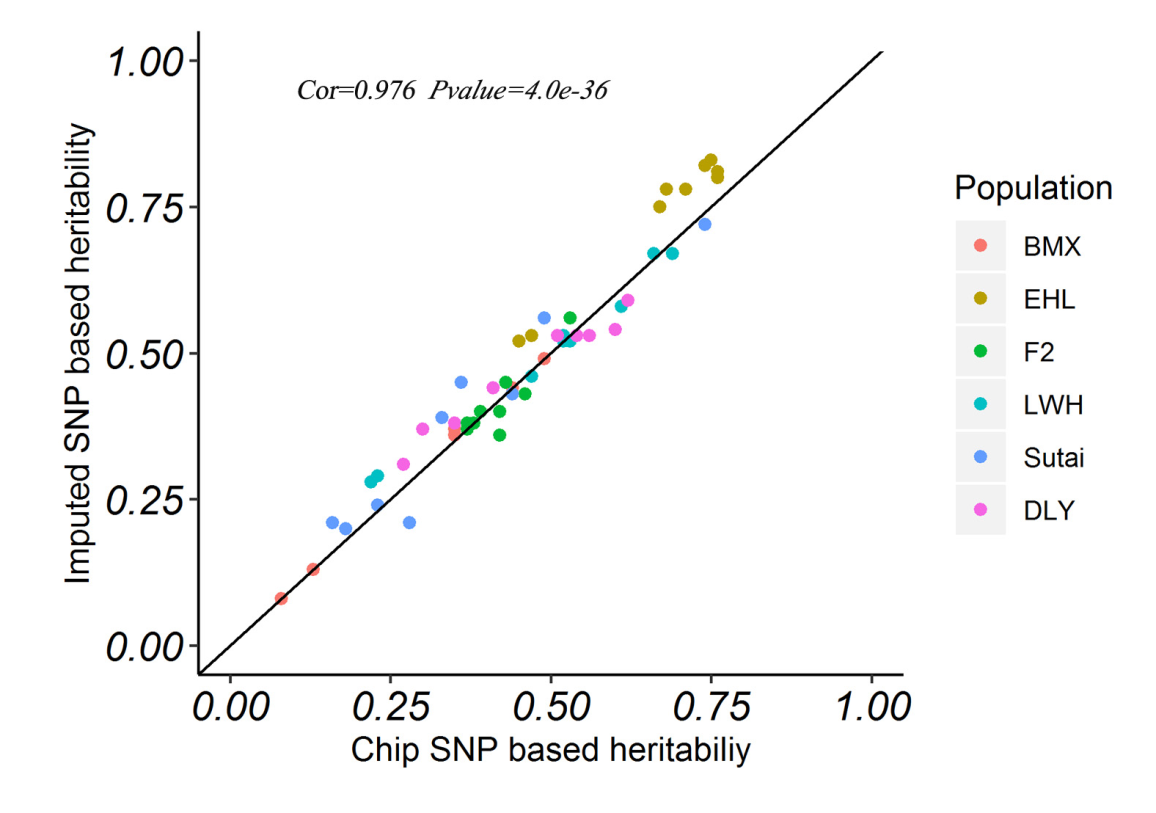


**Supplementary Figure S2**. Correlation of genomic heritability estimated from imputed SNPs and chip SNPs. Nine major fatty acid composition traits including C14:0, C16:0, C16:1n-7, C18:0, C18:1n-9, C18:2n-6, SFA, MUFA and PUFA were used in analysis.


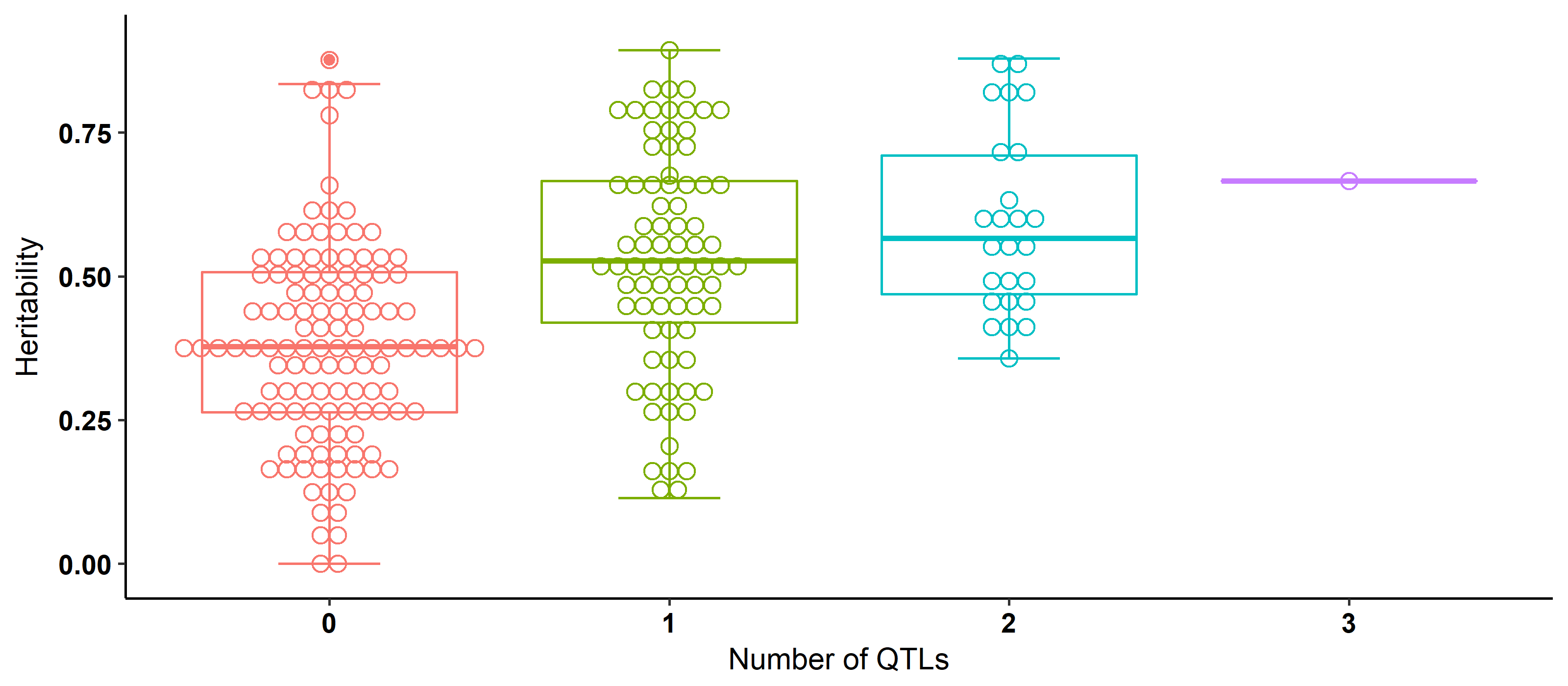


**Supplementary Figure S3.** The association between number of QTLs and heritability for the fatty acid composition traits in the six populations.

**1.2 Supplementary Tables**

**Supplementary Table S1**. Descriptive statistics of 38 fatty acid composition and metabolic traits in the six pig populations.

| **Trait** | **Full name** | **F_2_** | | | | **Sutai** | | | | **DLY** | | | |
| --- | --- | --- | --- | --- | --- | --- | --- | --- | --- | --- | --- | --- | --- |
|  |  | **N** | **Mean ± SD** | ***h*^2^ ± SE** | **λ** | **N** | **Mean ± SD** | ***h*^2^ ± SE** | **λ** | **N** | **Mean ± SD** | ***h*^2^ ± SE** | **λ** |
| C14:0 | Myristic acid | 590 | 1.097 ± 0.159 | 0.451 ± 0.064 | 1.049 | 294 | 1.458 ± 0.224 | 0.446 ± 0.114 | 1.027 | 608 | 1.311 ± 0.119 | 0.528 ± 0.083 | 0.997 |
| C16:0 | Palmitic acid | 591 | 23.540 ± 1.319 | 0.427 ± 0.062 | 1.084 | 293 | 25.541 ± 1.338 | 0.213 ± 0.131 | 1.010 | 608 | 23.881 ± 1.217 | 0.529 ± 0.080 | 1.022 |
| C18:0 | Stearic acid | 591 | 13.095 ± 1.193 | 0.402 ± 0.065 | 1.039 | 294 | 13.791 ± 1.564 | 0.723 ± 0.088 | 1.015 | 608 | 11.356 ± 1.138 | 0.541 ± 0.093 | 1.022 |
| C20:0 | Arachidic acid | 591 | 0.243 ± 0.067 | 0.446 ± 0.059 | 0.936 | 219 | 0.299 ± 0.064 | 0.715 ± 0.110 | 0.997 | 608 | 0.186 ± 0.032 | 0.727 ± 0.085 | 0.998 |
| C16:1n-7 | Palmitoleic acid | 591 | 3.004 ± 0.519 | 0.564 ± 0.054 | 1.009 | 295 | 3.137 ± 0.877 | 0.430 ± 0.098 | 1.040 | 608 | 3.905 ± 0.463 | 0.588 ± 0.092 | 1.029 |
| C18:1n-9 | Oleic acid | 591 | 44.380 ± 3.392 | 0.384 ± 0.060 | 1.045 | 290 | 43.269 ± 3.689 | 0.198 ± 0.105 | 1.056 | 608 | 45.363 ± 2.041 | 0.379 ± 0.085 | 1.009 |
| C20:1n-9 | Eicosenoic acid | 591 | 0.836 ± 0.178 | 0.509 ± 0.055 | 0.973 | 294 | 1.005 ± 0.225 | 0.401 ± 0.124 | 1.028 | 608 | 0.856 ± 0.115 | 0.449 ± 0.083 | 0.993 |
| C18:2n-6 | Linoleic acid | 591 | 8.922 ± 2.731 | 0.397 ± 0.062 | 1.068 | 292 | 8.329 ± 2.951 | 0.238 ± 0.113 | 1.011 | 603 | 5.349 ± 1.384 | 0.368 ± 0.100 | 1.006 |
| C18:3n-3 | α-linolenic acid | 589 | 0.189 ± 0.051 | 0.291 ± 0.057 | 1.016 | 204 | 0.232 ± 0.159 | 0.877 ± 0.083 | 1.014 | 604 | 0.191 ± 0.057 | 0.358 ± 0.100 | 1.016 |
| C20:2n-6 | Eicosadienoic acid | 591 | 0.435 ± 0.127 | 0.357 ± 0.062 | 1.051 | 281 | 0.376 ± 0.100 | 0.459 ± 0.113 | 0.991 | 607 | 0.278 ± 0.068 | 0.495 ± 0.097 | 1.016 |
| C20:3n-6 | Homolonolenic acid | 590 | 1.402 ± 0.822 | 0.273 ± 0.058 | 1.074 | 291 | 0.178 ± 0.124 | 0.309 ± 0.113 | 1.040 | 602 | 0.090 ± 0.032 | 0.150 ± 0.073 | 1.011 |
| C20:4n-6 | Arachidonic acid | 581 | 0.046 ± 0.011 | 0.181 ± 0.055 | 1.030 | 209 | 0.080 ± 0.067 | 0.658 ± 0.147 | 0.983 | 591 | 0.448 ± 0.214 | 0.175 ± 0.078 | 1.035 |
| C16:0/C14:0 | C16:0/C14:0 | 588 | 21.767 ± 2.548 | 0.310 ± 0.065 | 1.029 | 292 | 17.782 ± 2.319 | 0.417 ± 0.118 | 0.991 | 608 | 18.303 ± 1.216 | 0.443 ± 0.086 | 1.003 |
| C18:0/C16:0 | C18:0/C16:0 | 591 | 0.558 ± 0.054 | 0.481 ± 0.061 | 0.996 | 296 | 0.541 ± 0.061 | 0.636 ± 0.097 | 0.991 | 608 | 0.475 ± 0.039 | 0.566 ± 0.097 | 1.031 |
| C20:0/C18:0 | C20:0/C18:0 | 588 | 0.018 ± 0.005 | 0.490 ± 0.059 | 0.950 | 219 | 0.022 ± 0.004 | 0.861 ± 0.105 | 1.011 | 608 | 0.017 ± 0.003 | 0.776 ± 0.080 | 0.991 |
| C18:1n-9/C16:1n-7 | C18:1n-9/C16:1n-7 | 588 | 15.094 ± 2.439 | 0.655 ± 0.048 | 1.001 | 289 | 14.064 ± 3.396 | 0.629 ± 0.090 | 1.037 | 608 | 11.777 ± 1.488 | 0.608 ± 0.094 | 1.033 |
| C20:1n-9/C18:1n-9 | C20:1n-9/C18:1n-9 | 591 | 0.019 ± 0.004 | 0.488 ± 0.056 | 0.959 | 293 | 0.023 ± 0.005 | 0.381 ± 0.126 | 1.031 | 608 | 0.019 ± 0.003 | 0.503 ± 0.085 | 0.992 |
| C20:2n-6/C18:2n-6 | C20:2n-6/C18:2n-6 | 590 | 0.050 ± 0.010 | 0.160 ± 0.055 | 1.011 | 284 | 0.047 ± 0.008 | 0.308 ± 0.105 | 1.044 | 608 | 0.052 ± 0.006 | 0.258 ± 0.083 | 1.031 |
| C16:1n-7/C16:0 | C16:1n-7/C16:0 | 590 | 0.128 ± 0.021 | 0.501 ± 0.054 | 1.038 | 295 | 0.122 ± 0.034 | 0.504 ± 0.097 | 1.057 | 608 | 0.164 ± 0.021 | 0.602 ± 0.088 | 1.032 |
| C18:1n-9/C18:0 | C18:1n-9/C18:0 | 591 | 3.420 ± 0.433 | 0.411 ± 0.064 | 1.025 | 294 | 3.154 ± 0.518 | 0.590 ± 0.101 | 1.029 | 600 | 4.058 ± 0.499 | 0.547 ± 0.089 | 1.005 |
| C20:1n-9/C20:0 | C20:1n-9/C20:0 | 589 | 3.591 ± 0.879 | 0.469 ± 0.058 | 0.985 | 219 | 3.426 ± 0.906 | 0.443 ± 0.135 | 1.010 | 608 | 4.690 ± 0.808 | 0.490 ± 0.093 | 1.008 |
| C20:4n-6/C20:3n-6 | C20:4n-6/C20:3n-6 | 585 | 0.045 ± 0.027 | 0.178 ± 0.059 | 1.041 | 199 | 0.402 ± 0.293 | 0.449 ± 0.158 | 1.048 | 604 | 5.074 ± 1.313 | 0.158 ± 0.063 | 1.016 |
| C20:4n-6/C20:2n-6 | C20:4n-6/C20:2n-6 | 585 | 0.111 ± 0.024 | 0.166 ± 0.058 | 0.993 | 201 | 0.187 ± 0.136 | 0.527 ± 0.151 | 1.044 | 600 | 1.666 ± 0.728 | 0.095 ± 0.057 | 1.050 |
| C20:3n-6/C18:2n-6 | C20:3n-6/C18:2n-6 | 591 | 0.149 ± 0.053 | 0.233 ± 0.059 | 1.064 | 292 | 0.020 ± 0.009 | 0.517 ± 0.106 | 1.029 | 603 | 0.017 ± 0.004 | 0.173 ± 0.077 | 1.020 |
| C20:4n-6/C18:2n-6 | C20:4n-6/C18:2n-6 | 588 | 0.006 ± 0.002 | 0.193 ± 0.056 | 1.019 | 209 | 0.009 ± 0.006 | 0.452 ± 0.152 | 1.003 | 606 | 0.086 ± 0.033 | 0.053 ± 0.050 | 1.030 |
| n-3 | n-3 | 589 | 0.189 ± 0.051 | 0.291 ± 0.057 | 1.016 | 186 | 0.223 ± 3.379 | 0.935 ± 0.081 | 1.027 | 599 | 0.251 ± 0.073 | 0.255 ± 0.100 | 1.020 |
| n-6 | n-6 | 591 | 10.813 ± 3.564 | 0.375 ± 0.060 | 1.072 | 201 | 9.251 ± 0.161 | 0.223 ± 0.181 | 1.030 | 555 | 6.239 ± 1.666 | 0.264 ± 0.103 | 1.006 |
| n-6/n-3 | n-6/n-3 | 588 | 58.086 ± 17.374 | 0.269 ± 0.058 | 1.047 | 124 | 52.648 ± 45.398 | 0.354 ± 0.261 | 1.090 | 558 | 25.367 ± 5.122 | 0.000 ± 0.084 | 0.990 |
| SFA | SFA | 591 | 37.973 ± 2.040 | 0.357 ± 0.065 | 1.078 | 219 | 40.913 ± 2.433 | 0.561 ± 0.170 | 1.038 | 608 | 37.001 ± 2.164 | 0.526 ± 0.082 | 1.017 |
| MUFA | MUFA | 591 | 48.221 ± 3.609 | 0.371 ± 0.060 | 1.041 | 247 | 47.896 ± 3.727 | 0.205 ± 0.153 | 1.072 | 555 | 50.679 ± 2.113 | 0.439 ± 0.093 | 1.012 |
| PUFA | PUFA | 591 | 11.003 ± 3.589 | 0.377 ± 0.060 | 1.072 | 124 | 9.131 ± 2.900 | 0.385 ± 0.284 | 1.022 | 602 | 6.458 ± 1.706 | 0.31 ± 0.097 | 1.004 |
| MUFA/SFA | Ratio of MUFA to SFA | 590 | 1.272 ± 0.108 | 0.422 ± 0.065 | 1.043 | 180 | 1.168 ± 0.136 | 0.270 ± 0.178 | 1.039 | 555 | 1.373 ± 0.119 | 0.569 ± 0.089 | 1.002 |
| PUFA/SFA | Ratio of PUFA to SFA | 591 | 0.293 ± 0.105 | 0.372 ± 0.060 | 1.078 | 111 | 0.228 ± 0.078 | 0.179 ± 0.276 | 1.055 | 604 | 0.177 ± 0.053 | 0.341 ± 0.092 | 1.006 |
| ACL | Average Chain Length | 581 | 17.003 ± 0.178 | 0.161 ± 0.054 | 1.053 | 79 | 17.011 ± 0.114 | 0.332 ± 0.358 | 1.072 | 517 | 16.410 ± 0.156 | 0.114 ± 0.084 | 1.028 |
| DBI | Double-bond indices | 591 | 0.719 ± 0.052 | 0.383 ± 0.062 | 1.081 | 107 | 0.664 ± 0.038 | 0.264 ± 0.283 | 1.086 | 525 | 0.650 ± 0.035 | 0.347 ± 0.094 | 1.024 |
| UI | Unsaturated indices | 591 | 1.438 ± 0.105 | 0.383 ± 0.062 | 1.081 | 107 | 1.328 ± 0.076 | 0.264 ± 0.283 | 1.086 | 525 | 1.300 ± 0.070 | 0.347 ± 0.094 | 1.024 |
| FattyAI | Fatty acid atherogenic index | 591 | 0.472 ± 0.040 | 0.519 ± 0.060 | 1.070 | 106 | 0.548 ± 0.051 | 0.001 ± 0.214 | 1.112 | 554 | 0.512 ± 0.043 | 0.576 ± 0.088 | 1.026 |
| FattyTI | Fatty acid thrombogenic index | 590 | 1.255 ± 0.100 | 0.413 ± 0.065 | 1.080 | 107 | 0.585 ± 0.261 | 0.291 ± 0.244 | 1.045 | 526 | 1.255 ± 0.117 | 0.551 ± 0.092 | 1.027 |

| **Laiwu** | | | | **Erhualian** | | | | **Bamaxiang** | | | |
| --- | --- | --- | --- | --- | --- | --- | --- | --- | --- | --- | --- |
| **N** | **Mean ± SD** | ***h*^2^ ± SE** | **λ** | **N** | **Mean ± SD** | ***h*^2^ ± SE** | **λ** | **N** | **Mean ± SD** | ***h*^2^ ± SE** | **λ** |
| 280 | 1.332 ± 0.120 | 0.669 ± 0.094 | 0.983 | 331 | 1.337 ± 0.178 | 0.835 ± 0.080 | 0.993 | 315 | 1.249 ± 0.295 | 0.082 ± 0.063 | 1.011 |
| 280 | 26.544 ± 0.992 | 0.527 ± 0.088 | 1.005 | 331 | 25.003 ± 1.300 | 0.746 ± 0.085 | 1.002 | 315 | 21.977 ± 1.211 | 0.127 ± 0.074 | 1.026 |
| 278 | 12.053 ± 1.006 | 0.578 ± 0.092 | 1.032 | 331 | 12.579 ± 1.249 | 0.800 ± 0.081 | 1.018 | 315 | 14.693 ± 1.451 | 0.438 ± 0.112 | 1.011 |
| 280 | 0.193 ± 0.026 | 0.633 ± 0.112 | 0.989 | 331 | 0.247 ± 0.046 | 0.791 ± 0.078 | 0.973 | 315 | 0.382 ± 0.073 | 0.459 ± 0.132 | 0.972 |
| 280 | 3.854 ± 0.603 | 0.675 ± 0.069 | 1.009 | 331 | 3.618 ± 0.678 | 0.813 ± 0.082 | 0.989 | 315 | 2.928 ± 0.533 | 0.199 ± 0.118 | 1.007 |
| 280 | 46.367 ± 1.611 | 0.518 ± 0.105 | 1.011 | 331 | 46.167 ± 2.263 | 0.777 ± 0.087 | 0.998 | 315 | 46.576 ± 2.07 | 0.371 ± 0.103 | 1.005 |
| 280 | 0.752 ± 0.149 | 0.804 ± 0.067 | 0.971 | 331 | 0.737 ± 0.127 | 0.666 ± 0.113 | 0.954 | 315 | 1.704 ± 0.306 | 0.310 ± 0.171 | 1.016 |
| 277 | 3.595 ± 0.584 | 0.294 ± 0.117 | 1.002 | 331 | 4.465 ± 0.893 | 0.534 ± 0.101 | 1.016 | 315 | 3.952 ± 0.836 | 0.363 ± 0.101 | 1.015 |
| 278 | 0.112 ± 0.025 | 0.118 ± 0.096 | 1.031 | 330 | 0.132 ± 0.030 | 0.525 ± 0.100 | 1.030 | 315 | 0.125 ± 0.034 | 0.251 ± 0.094 | 1.016 |
| 278 | 0.195 ± 0.033 | 0.419 ± 0.120 | 0.976 | 330 | 0.252 ± 0.053 | 0.653 ± 0.099 | 1.020 | 315 | 0.371 ± 0.077 | 0.510 ± 0.103 | 0.995 |
| 276 | 0.050 ± 0.011 | 0.589 ± 0.089 | 1.003 | 330 | 0.077 ± 0.018 | 0.507 ± 0.122 | 0.990 | 313 | 0.106 ± 0.031 | 0.438 ± 0.107 | 1.005 |
| 275 | 0.176 ± 0.050 | 0.582 ± 0.095 | 1.007 | 330 | 0.251 ± 0.081 | 0.387 ± 0.123 | 1.029 | 310 | 0.393 ± 0.155 | 0.294 ± 0.109 | 1.011 |
| 305 | 19.963 ± 1.372 | 0.612 ± 0.100 | 0.990 | 331 | 18.943 ± 1.955 | 0.763 ± 0.090 | 0.989 | 315 | 18.425 ± 3.726 | 0.185 ± 0.083 | 1.009 |
| 303 | 0.455 ± 0.037 | 0.600 ± 0.079 | 1.009 | 331 | 0.504 ± 0.053 | 0.764 ± 0.086 | 1.005 | 315 | 0.671 ± 0.080 | 0.189 ± 0.101 | 1.016 |
| 278 | 0.016 ± 0.002 | 0.674 ± 0.118 | 1.002 | 331 | 0.020 ± 0.003 | 0.789 ± 0.085 | 0.950 | 315 | 0.026 ± 0.004 | 0.491 ± 0.122 | 0.969 |
| 304 | 12.287 ± 2.064 | 0.599 ± 0.076 | 1.017 | 330 | 13.269 ± 3.073 | 0.814 ± 0.083 | 0.978 | 315 | 16.446 ± 3.146 | 0.167 ± 0.108 | 1.011 |
| 280 | 0.016 ± 0.003 | 0.791 ± 0.071 | 0.972 | 330 | 0.016 ± 0.003 | 0.595 ± 0.120 | 0.958 | 315 | 0.037 ± 0.007 | 0.289 ± 0.182 | 1.023 |
| 280 | 0.054 ± 0.007 | 0.737 ± 0.088 | 0.995 | 331 | 0.057 ± 0.007 | 0.609 ± 0.107 | 0.990 | 315 | 0.095 ± 0.015 | 0.296 ± 0.169 | 1.018 |
| 305 | 0.146 ± 0.022 | 0.645 ± 0.074 | 1.022 | 331 | 0.145 ± 0.025 | 0.828 ± 0.078 | 0.998 | 315 | 0.133 ± 0.022 | 0.279 ± 0.114 | 1.009 |
| 305 | 3.876 ± 0.410 | 0.566 ± 0.094 | 1.024 | 331 | 3.713 ± 0.463 | 0.826 ± 0.075 | 1.030 | 315 | 3.204 ± 0.370 | 0.606 ± 0.111 | 1.011 |
| 280 | 3.937 ± 0.753 | 0.894 ± 0.049 | 0.985 | 331 | 3.050 ± 0.595 | 0.879 ± 0.068 | 0.977 | 315 | 4.560 ± 0.917 | 0.475 ± 0.117 | 0.995 |
| 278 | 3.583 ± 0.802 | 0.510 ± 0.107 | 1.013 | 331 | 3.285 ± 0.752 | 0.538 ± 0.109 | 1.017 | 313 | 3.749 ± 0.965 | 0.401 ± 0.124 | 0.994 |
| 277 | 0.916 ± 0.241 | 0.441 ± 0.102 | 1.012 | 329 | 1.003 ± 0.285 | 0.437 ± 0.109 | 1.014 | 310 | 1.074 ± 0.388 | 0.256 ± 0.115 | 1.021 |
| 279 | 0.014 ± 0.003 | 0.381 ± 0.103 | 1.013 | 330 | 0.017 ± 0.003 | 0.528 ± 0.105 | 0.965 | 312 | 0.027 ± 0.006 | 0.560 ± 0.124 | 1.008 |
| 277 | 0.049 ± 0.012 | 0.362 ± 0.101 | 1.014 | 330 | 0.057 ± 0.015 | 0.424 ± 0.106 | 1.025 | 314 | 0.101 ± 0.032 | 0.363 ± 0.130 | 1.007 |
| 303 | 0.138 ± 0.029 | 0.046 ± 0.075 | 1.033 | 329 | 0.159 ± 0.036 | 0.546 ± 0.099 | 1.029 | 315 | 0.125 ± 0.034 | 0.251 ± 0.094 | 1.016 |
| 289 | 4.022 ± 0.646 | 0.290 ± 0.111 | 0.998 | 328 | 5.075 ± 0.998 | 0.517 ± 0.105 | 1.018 | 315 | 4.836 ± 1.044 | 0.359 ± 0.098 | 1.013 |
| 281 | 28.947 ± 3.613 | 0.274 ± 0.128 | 1.033 | 326 | 32.162 ± 2.792 | 0.480 ± 0.109 | 1.028 | 315 | 40.035 ± 7.701 | 0.131 ± 0.104 | 1.009 |
| 305 | 40.221 ± 1.718 | 0.516 ± 0.092 | 1.027 | 331 | 39.257 ± 2.097 | 0.816 ± 0.077 | 1.017 | 315 | 38.392 ± 1.871 | 0.489 ± 0.103 | 1.018 |
| 302 | 51.172 ± 1.54 | 0.461 ± 0.098 | 1.017 | 330 | 50.691 ± 2.116 | 0.780 ± 0.086 | 1.016 | 315 | 51.298 ± 2.029 | 0.436 ± 0.107 | 1.002 |
| 289 | 4.160 ± 0.664 | 0.277 ± 0.112 | 0.999 | 327 | 5.236 ± 1.033 | 0.516 ± 0.105 | 1.019 | 315 | 4.960 ± 1.066 | 0.359 ± 0.098 | 1.013 |
| 302 | 1.276 ± 0.087 | 0.568 ± 0.089 | 1.029 | 331 | 1.297 ± 0.118 | 0.835 ± 0.075 | 1.026 | 315 | 1.341 ± 0.109 | 0.510 ± 0.108 | 1.012 |
| 289 | 0.104 ± 0.018 | 0.387 ± 0.107 | 0.998 | 327 | 0.134 ± 0.027 | 0.526 ± 0.107 | 1.018 | 315 | 0.130 ± 0.029 | 0.378 ± 0.097 | 1.022 |
| 292 | 16.577 ± 0.123 | 0.482 ± 0.101 | 1.020 | 288 | 16.544 ± 0.148 | 0.710 ± 0.102 | 1.007 | 315 | 16.547 ± 0.146 | 0.143 ± 0.101 | 1.018 |
| 290 | 0.602 ± 0.019 | 0.520 ± 0.097 | 1.015 | 327 | 0.619 ± 0.023 | 0.658 ± 0.098 | 1.007 | 315 | 0.623 ± 0.022 | 0.429 ± 0.099 | 1.021 |
| 290 | 1.204 ± 0.038 | 0.520 ± 0.097 | 1.015 | 327 | 1.238 ± 0.047 | 0.658 ± 0.098 | 1.007 | 315 | 1.628 ± 0.128 | 0.511 ± 0.103 | 1.014 |
| 292 | 0.575 ± 0.038 | 0.570 ± 0.091 | 1.010 | 327 | 0.545 ± 0.051 | 0.820 ± 0.082 | 0.999 | 315 | 0.481 ± 0.050 | 0.155 ± 0.076 | 1.032 |
| 292 | 1.421 ± 0.098 | 0.515 ± 0.095 | 1.027 | 327 | 1.376 ± 0.118 | 0.814 ± 0.079 | 1.018 | 315 | 1.335 ± 0.103 | 0.484 ± 0.102 | 1.011 |

**Supplementary Table S2.** Summary information on the 396 sequenced individuals.

| **Groups** | **Breeds (sample size)** | **N** | **Coverage** | **Data sources** |
| --- | --- | --- | --- | --- |
|  |  |  |  |  |
| Eastern China | Erhualian (29), Jinhua (9), Jiangquhai (1), Meishan (10) | 49 | 25 | JXAU |
| Southern China | Bamaxiang (12), Wuzhishan (6), Luchuan (6), Xiang (2) | 26 | 25 | JXAU |
| Southwest China | Pengzhou (3), Bamei (6),  Neijiang (9), Baoshan (6) | 24 | 25 | JXAU |
| Northern China | Laiwu (15), Min (6), Hetao (6) | 27 | 25 | JXAU |
| Middle China | Tongcheng (4) | 4 | 7 | HZAU |
| High altitude region in China | Yunan Tibetan (17), Tibet  Tibetan (22), Sichuan Tibetan (29), Gansu Tibetan (23) | 91 | 25, 5 | JXAU,  SCAU ([Li et al., 2013](#_ENREF_2)) |
|  |  |  |  |  |
| International Commercial breeds | White Duroc (10), Duroc (22), Landrace (24), Large White (71), Pietrain (11), Hampshire (2), | 140 | ~25,8 | JXAU,  WAU ([Groenen et al., 2012](#_ENREF_1)),  Korea ([Moon et al., 2015](#_ENREF_3)) |
|  |  |  |  |  |
| Wild Boars | Asian wild boar (17),  European wild boar (18) | 35 | 25, 8, 5 | JXAU, WAU, SCAU |

**Supplementary Table S3**. Summary of significant loci (*p*-value < 5 × 10^-8^) identified for fatty acid composition and metabolism traits in single population GWAS.

| **chromosome:** | **Trait** | **Pop** | **Candidate**  **Genes** | ***p-*value** | **MAF** | **Var** | **Variant** | **Overlap** | **Overlap** | **Category** | **Increased** | **Positions of lead** |
| --- | --- | --- | --- | --- | --- | --- | --- | --- | --- | --- | --- | --- |
| **position** |  |  |  |  | **(minor/major)** | **(%)** | **Annotation** | **with enhancer** | **with promoter** |  | **Significance (unit)** | **SNPs on *Sscrofa* 11.1** |
| 1:8873447 | C18:0 | Bamaxiang | *-* | 3.02E-08 | 0.30 (A/G) | 11.24 | Intron | - | - | Novel | - | 1:7195408 |
| 2:9261581 | C20:3n-6/C18:2n-6 | Bamaxiang | *FADS1* | 2.10E-10 | 0.40 (G/A) | 17.83 | Upstream | Liver_H3K27Ac | Liver_H3K4me3 | Replicated | - | 2:9736686 |
| 2:9687907 | C20:4n-6/C20:3n-6 | Bamaxiang | *FADS1* | 2.76E-10 | 0.49 (T/C) | 12.21 | Upstream | Liver_H3K27Ac | - | Replicated | - | 2:10140593 |
| 2:145804018 | FattyAI | Bamaxiang | *-* | 5.09E-09 | 0.38 (T/C) | 12.50 | Intergenic | - | - | Novel | - | 2:140058025 |
| 7:30614484 | C18:1n-9/C16:1n-7 | Bamaxiang | *-* | 3.40E-09 | 0.12 (C/T) | 11.85 | Upstream | - | - | Novel | - | 7:26146752 |
| 7:52837555 | C20:1n-9 | Bamaxiang | *ACSBG1* | 8.21E-10 | 0.24 (G/A) | 11.69 | Intergenic | - | - | Replicated | - | 7:47330441 |
| 7:52877903 | C20:1n-9/C18:1n-9 | Bamaxiang | *ACSBG1* | 1.87E-09 | 0.16 (A/G) | 10.28 | Intergenic | - | - | Replicated | - | 7:47290117 |
| 8:138708016 | C20:4n-6/C20:2n-6 | Bamaxiang | *SNCA* | 3.14E-08 | 0.06 (A/G) | 9.13 | Intron | - | - | Novel | - | 8:129321928 |
| 13:40365857 | ACL | Bamaxiang | *ACOX2* | 4.00E-09 | 0.38 (T/G) | 11.35 | Intergenic | - | - | Replicated | - | 13:37034334 |
| 16:42563988 | C20:0 | Bamaxiang | *ELOVL7* | 6.10E-20 | 0.20 (A/G) | 28.61 | Intron | - | - | Replicated | - | 16:39654025 |
| 16:42563988 | C20:0/C18:0 | Bamaxiang | *ELOVL7* | 7.82E-34 | 0.20 (A/G) | 45.72 | Intron | - | - | Replicated | - | 16:39654025 |
| 16:48557856 | C20:1n-9/C20:0 | Bamaxiang | *-* | 3.10E-11 | 0.38 (G/A) | 17.33 | Intergenic | - | - | Novel | - | 16:44950249 |
| 2:115831690 | C20:2n-6 | DLY | *-* | 5.22E-09 | 0.06 (C/A) | 5.20 | Intergenic | - | - | Novel | - | 2:111472105 |
| 7:52762692 | C20:2n-6/C18:2n-6 | DLY | *ACSBG1* | 1.57E-09 | 0.42 (C/A) | 5.86 | Intergenic | - | - | Replicated | - | 7:47316913 |
| 7:52771142 | C20:1n-9 | DLY | *ACSBG1* | 2.68E-08 | 0.07 (G/A) | 6.59 | Intron | - | - | Replicated | - | 7:47397239 |
| 7:52771142 | C20:1n-9/C18:1n-9 | DLY | *ACSBG1* | 2.93E-08 | 0.07 (G/A) | 6.73 | Intron | - | - | Replicated | - | 7:47397239 |
| 8:119726275 | C16:1n-7 | DLY | *ELOVL6* | 3.04E-12 | 0.06 (T/C) | 8.39 | Intergenic | - | - | Replicated | - | 8:111428042 |
| 8:119726275 | C16:1n-7/C16:0 | DLY | *ELOVL6* | 4.34E-08 | 0.06 (T/C) | 5.12 | Intergenic | - | - | Replicated | - | 8:111428042 |
| 8:119726275 | C18:1n-9/C16:1n-7 | DLY | *ELOVL6* | 1.90E-18 | 0.06 (T/C) | 13.06 | Intergenic | - | - | Replicated | - | 8:111428042 |
| 8:119939409 | C18:0/C16:0 | DLY | *ELOVL6* | 8.88E-09 | 0.10 (T/C) | 6.26 | Intergenic | - | - | Replicated | - | 8:111736573 |
| 8:129588470 | ACL | DLY | *MTTP* | 1.64E-08 | 0.12 (G/A) | 6.35 | Intergenic | - | - | Novel | - | 8:120408885 |
| 14:121409756 | C18:1n-9/C16:1n-7 | DLY | *SCD* | 5.44E-10 | 0.31 (T/C) | 6.79 | Intergenic | - | - | Replicated | - | 14:111750123 |
| 14:121425536 | C16:0 | DLY | *SCD* | 1.92E-09 | 0.39 (A/G) | 9.09 | Intergenic | - | - | Replicated | - | 14:111765859 |
| 14:121444696 | C18:0/C16:0 | DLY | *SCD* | 7.01E-25 | 0.33 (G/A) | 21.48 | Intergenic | - | - | Replicated | - | 14:111785114 |
| 14:121445411 | SFA | DLY | *SCD* | 9.03E-24 | 0.33 (T/C) | 21.68 | Intergenic | - | - | Enhanced | 2 | 14:111785822 |
| 14:121445488 | MUFA/SFA | DLY | *SCD* | 5.78E-22 | 0.33 (G/T) | 23.52 | Intergenic | - | - | Replicated | - | 14:111785899 |
| 14:121446694 | FattyTI | DLY | *SCD* | 3.59E-20 | 0.37 (G/A) | 21.34 | Intergenic | - | - | Replicated | - | 14:111787102 |
| 14:121454019 | C18:0 | DLY | *SCD* | 3.14E-33 | 0.33 (T/G) | 28.20 | Intergenic | - | - | Enhanced | 3 | 14:111794422 |
| 14:121454019 | C18:1n-9/C18:0 | DLY | *SCD* | 2.70E-29 | 0.33 (T/G) | 27.26 | Intergenic | - | - | Enhanced | 2 | 14:111794422 |
| 14:121469254 | FattyAI | DLY | *SCD* | 3.03E-12 | 0.36 (G/A) | 13.24 | Intergenic | - | - | Replicated | - | 14:111811103 |
| 14:121505487 | C16:1n-7 | DLY | *SCD* | 5.27E-15 | 0.33 (G/T) | 12.25 | Intron | - | - | Replicated | - | 14:111847266 |
| 14:121505487 | C16:1n-7/C16:0 | DLY | *SCD* | 3.17E-21 | 0.33 (G/T) | 18.35 | Intron | - | - | Replicated | - | 14:111847266 |
| 14:121664759 | DBI | DLY | *SCD* | 1.22E-10 | 0.34 (C/T) | 9.19 | Intron | Liver_H3K27Ac | - | Enhanced | 2 | 14:112056813 |
| 14:121664759 | UI | DLY | *SCD* | 1.22E-10 | 0.34 (C/T) | 9.19 | Intron | Liver_H3K27Ac | - | Enhanced | 2 | 14:112056813 |
| 14:121931507 | MUFA | DLY | *SCD* | 1.74E-11 | 0.36 (T/C) | 14.56 | Intergenic | - | - | Replicated | - | 14:112201173 |
| 16:41442373 | C20:1n-9 | DLY | *ELOVL7* | 3.13E-08 | 0.05 (G/A) | 5.75 | Intergenic | - | - | Replicated | - | 16:38491214 |
| 16:43497948 | C20:0/C18:0 | DLY | *ELOVL7* | 3.66E-62 | 0.12 (A/T) | 43.50 | Intergenic | Liver_H3K27Ac | - | Enhanced | 3 | 16:40497914 |
| 16:43506001 | C20:0 | DLY | *ELOVL7* | 2.19E-45 | 0.12 (G/A) | 33.64 | Intergenic | - | - | Enhanced | 4 | 16:40505966 |
| 16:43561167 | C20:1n-9/C18:1n-9 | DLY | *ELOVL7* | 2.11E-08 | 0.13 (G/A) | 6.37 | Intergenic | - | - | Replicated | - | 16:40561137 |
| 16:43561222 | C20:1n-9/C20:0 | DLY | *ELOVL7* | 5.66E-17 | 0.13 (T/G) | 14.37 | Intergenic | - | - | Replicated | - | - |
| 2:746298 | ACL | Erhualian | *-* | 2.31E-08 | 0.07 (G/A) | 17.70 | Upstream | Liver_H3K27Ac | Liver_H3K4me3 | Novel | - | - |
| 2:8927158 | C20:3n-6 | Erhualian | *FADS2* | 2.28E-09 | 0.49 (A/G) | 9.02 | Intergenic | - | - | Replicated | - | 2:9479147 |
| 2:8929954 | C20:3n-6/C18:2n-6 | Erhualian | *FADS2* | 2.90E-22 | 0.43 (C/T) | 25.05 | Intergenic | - | - | Enhanced | 4 | 2:9476353 |
| 2:9080952 | C20:4n-6/C20:3n-6 | Erhualian | *FADS2* | 1.15E-11 | 0.30 (G/C) | 22.45 | Intergenic | - | - | Replicated | - | 2:9705017 |
| 3:92010559 | C20:1n-9 | Erhualian | *-* | 9.96E-09 | 0.33 (T/C) | 11.77 | Intergenic | - | - | Novel | - | 3:42352506 |
| 7:134678195 | C20:1n-9/C18:1n-9 | Erhualian | *ELOVL5* | 1.07E-23 | 0.22 (G/T) | 34.48 | Intergenic | - | - | Replicated | - | 7:46997388 |
| 7:134690924 | C20:1n-9/C20:0 | Erhualian | *ELOVL5* | 4.62E-19 | 0.41 (G/A) | 26.49 | Intergenic | - | - | Replicated | - | 7:46984756 |
| 7:134695178 | C20:2n-6/C18:2n-6 | Erhualian | *ELOVL5* | 1.21E-14 | 0.42 (C/T) | 18.27 | Intergenic | Liver_H3K27Ac | - | Replicated | - | 7:46980501 |
| 7:134705405 | C20:1n-9 | Erhualian | *ELOVL5* | 8.37E-22 | 0.27 (C/T) | 31.14 | Intergenic | - | - | Replicated | - | 7:46970274 |
| 8:120185979 | C16:1n-7 | Erhualian | *ELOVL6* | 2.48E-10 | 0.36 (A/G) | 21.23 | Intron | - | - | Replicated | - | 8:112072835 |
| 8:120185979 | C16:1n-7/C16:0 | Erhualian | *ELOVL6* | 1.11E-08 | 0.36 (A/G) | 19.62 | Intron | - | - | Replicated | - | 8:112072835 |
| 8:120185979 | C18:1n-9/C16:1n-7 | Erhualian | *ELOVL6* | 1.61E-10 | 0.36 (A/G) | 18.06 | Intron | - | - | Replicated | - | 8:112072835 |
| 9:11302313 | C20:4n-6/C20:3n-6 | Erhualian | *DGAT2* | 3.13E-08 | 0.07 (G/A) | 7.30 | Intron | - | - | Novel | - | 9:10212537 |
| 12:387430 | C20:1n-9 | Erhualian | *FASN* | 3.98E-12 | 0.23 (T/C) | 12.86 | Intron | - | - | Replicated | - | 12:350246 |
| 12:1176481 | C16:1n-7 | Erhualian | *FASN* | 8.67E-20 | 0.07 (T/C) | 25.34 | Intergenic | - | - | Enhanced | 2 | 12:1333661 |
| 12:1176481 | C18:1n-9/C16:1n-7 | Erhualian | *FASN* | 2.38E-30 | 0.07 (T/C) | 40.86 | Intergenic | - | - | Enhanced | 4 | 12:1333661 |
| 12:1176481 | C16:0 | Erhualian | *FASN* | 1.01E-12 | 0.07 (T/C) | 28.19 | Intergenic | - | - | Replicated | - | 12:1333661 |
| 12:1176481 | C16:1n-7/C16:0 | Erhualian | *FASN* | 1.23E-16 | 0.07 (T/C) | 18.37 | Intergenic | - | - | Replicated | - | 12:1333661 |
| 12:1381215 | C18:1n-9 | Erhualian | *FASN* | 1.85E-11 | 0.29 (C/T) | 18.92 | Intron | - | - | Replicated | - | 12:1487861 |
| 12:1482194 | C14:0 | Erhualian | *FASN* | 1.09E-21 | 0.24 (G/A) | 30.47 | Intron | - | - | Replicated | - | 12:1576644 |
| 12:1482194 | C16:0/C14:0 | Erhualian | *FASN* | 3.41E-22 | 0.24 (G/A) | 32.80 | Intron | - | - | Replicated | - | 12:1576644 |
| 12:1485619 | FattyAI | Erhualian | *FASN* | 3.58E-15 | 0.29 (C/T) | 27.64 | Intron | - | - | Replicated | - | 12:1580069 |
| 12:1565371 | DBI | Erhualian | *FASN* | 1.12E-10 | 0.07 (G/C) | 21.43 | Intron | Liver_H3K27Ac | - | Replicated | - | 12:1659672 |
| 12:1565371 | UI | Erhualian | *FASN* | 1.12E-10 | 0.07 (G/C) | 21.43 | Intron | Liver_H3K27Ac | - | Replicated | - | 12:1659672 |
| 12:1569409 | C18:0/C16:0 | Erhualian | *FASN* | 1.08E-15 | 0.08 (A/G) | 14.65 | Intron | Liver_H3K27Ac | - | Enhanced | 5 | 12:1663708 |
| 12:1569409 | ACL | Erhualian | *FASN* | 9.47E-13 | 0.08 (A/G) | 20.94 | Intron | Liver_H3K27Ac | - | Replicated | - | 12:1663708 |
| 12:1569490 | C20:2n-6 | Erhualian | *FASN* | 1.17E-08 | 0.07 (A/G) | 9.40 | Intron | Liver_H3K27Ac | - | Replicated | - | 12:1663789 |
| 12:56801697 | C18:0 | Erhualian | *-* | 1.02E-09 | 0.06 (T/C) | 14.43 | Upstream | - | - | Novel | - | 12:54049222 |
| 12:57421524 | SFA | Erhualian | *-* | 5.01E-09 | 0.10 (T/C) | 18.68 | Upstream | - | - | Novel | - | 12:54663378 |
| 16:42561841 | C20:0 | Erhualian | *ELOVL7* | 8.25E-30 | 0.48 (G/A) | 41.84 | Intron | - | - | Replicated | - | 16:39651879 |
| 16:42561841 | C20:0/C18:0 | Erhualian | *ELOVL7* | 3.77E-44 | 0.48 (G/A) | 50.47 | Intron | - | - | Replicated | - | 16:39651879 |
| 16:42561841 | C20:1n-9/C20:0 | Erhualian | *ELOVL7* | 1.37E-13 | 0.48 (G/A) | 22.21 | Intron | - | - | Replicated | - | 16:39651879 |
| 2:74664653 | C20:3n-6 | F_2_ | *PLIN5* | 1.89E-08 | 0.06 (G/A) | 4.66 | Intergenic | Liver_H3K27Ac | - | Novel | - | 2:73882468 |
| 4:63910717 | C18:1n-9/C18:0 | F_2_ | *-* | 1.69E-10 | 0.14 (A/G) | 9.49 | Intron | - | Liver_H3K4me3 | Replicated | - | 4:111632724 |
| 4:63923829 | MUFA/SFA | F_2_ | *-* | 6.76E-10 | 0.14 (G/A) | 9.27 | Intron | - | - | Replicated | - | 4:111619429 |
| 4:63986437 | C18:0 | F_2_ | *-* | 1.72E-09 | 0.14 (G/A) | 7.86 | Intergenic | - | - | Replicated | - | - |
| 4:86701762 | C20:2n-6 | F_2_ | *-* | 6.22E-09 | 0.42 (G/T) | 9.30 | Intergenic | - | - | Novel | - | 4:79286302 |
| 7:31286841 | C20:4n-6/C20:2n-6 | F_2_ | *-* | 1.36E-08 | 0.46 (T/A) | 7.06 | Intergenic | - | - | Replicated | - | 7:26844436 |
| 7:31959362 | C18:0/C16:0 | F_2_ | *-* | 5.58E-09 | 0.46 (A/G) | 9.12 | Downstream | - | - | Replicated | - | 7:27532685 |
| 7:34710056 | C20:2n-6 | F_2_ | *-* | 3.08E-10 | 0.46 (A/G) | 7.77 | Intergenic | - | - | Enhanced | 2 | 7:30050265 |
| 7:34919339 | C18:3n-3 | F_2_ | *-* | 4.93E-15 | 0.48 (A/G) | 15.04 | Intron | - | - | Replicated | - | 7:30259194 |
| 7:34919339 | n-3 | F_2_ | *-* | 4.93E-15 | 0.48 (A/G) | 15.04 | Intron | - | - | Replicated | - | 7:30259194 |
| 7:134527363 | C20:1n-9 | F_2_ | *ELOVL5* | 1.58E-23 | 0.43 (C/G) | 31.18 | Intergenic | - | - | Enhanced | 10 | 7:46810502 |
| 7:134527363 | C20:1n-9/C18:1n-9 | F_2_ | *ELOVL5* | 4.98E-23 | 0.43 (C/G) | 28.55 | Intergenic | - | - | Enhanced | 10 | 7:46810502 |
| 7:134556509 | C20:1n-9/C20:0 | F_2_ | *ELOVL5* | 7.14E-11 | 0.42 (C/T) | 15.38 | Intergenic | - | - | Enhanced | 2 | 7:46824994 |
| 8:120599982 | C18:1n-9/C16:1n-7 | F_2_ | *ELOVL6* | 3.99E-12 | 0.41 (G/A) | 9.65 | Downstream | - | - | Novel | - | 8:112645361 |
| 8:120605047 | C16:1n-7 | F_2_ | *ELOVL6* | 1.19E-09 | 0.42 (C/G) | 6.97 | Intergenic | Liver_H3K27Ac | Liver_H3K4me3 | Enhanced | 3 | 8:112651294 |
| 8:120605047 | C16:1n-7/C16:0 | F_2_ | *ELOVL6* | 3.98E-08 | 0.42 (C/G) | 6.20 | Intergenic | Liver_H3K27Ac | Liver_H3K4me3 | Novel | - | 8:112651294 |
| 14:120092295 | C18:1n-9/C18:0 | F_2_ | *SCD* | 8.04E-14 | 0.15 (C/T) | 11.85 | Intergenic | - | - | Enhanced | 2 | 14:110647236 |
| 14:120092725 | MUFA/SFA | F_2_ | *SCD* | 4.32E-14 | 0.15 (G/A) | 12.04 | Intergenic | - | - | Enhanced | 4 | 14:110647666 |
| 14:120175235 | FattyTI | F_2_ | *SCD* | 9.59E-09 | 0.17 (G/A) | 6.75 | Intergenic | Liver_H3K27Ac | - | Enhanced | 2 | 14:110729335 |
| 14:120287737 | C18:0/C16:0 | F_2_ | *SCD* | 1.25E-08 | 0.37 (A/G) | 9.55 | Intron | - | - | Replicated | - | 14:110841408 |
| 14:120348690 | C16:1n-7 | F_2_ | *SCD* | 5.64E-09 | 0.15 (T/C) | 7.60 | Intron | - | - | Enhanced | 3 | - |
| 14:120348690 | C16:1n-7/C16:0 | F_2_ | *SCD* | 4.74E-11 | 0.15 (T/C) | 8.69 | Intron | - | - | Enhanced | 4 | - |
| 14:121458036 | C18:0 | F_2_ | *SCD* | 2.04E-12 | 0.16 (G/A) | 9.96 | Intergenic | - | - | Enhanced | 2 | 14:111799839 |
| 16:41383845 | C20:1n-9/C20:0 | F_2_ | *ELOVL7* | 9.08E-26 | 0.29 (G/A) | 22.73 | Intergenic | - | - | Replicated | - | 16:38545772 |
| 16:41393886 | C20:0/C18:0 | F_2_ | *ELOVL7* | 3.94E-44 | 0.29 (A/G) | 36.26 | Intergenic | - | - | Replicated | - | 16:38557437 |
| 16:41430191 | C20:0 | F_2_ | *ELOVL7* | 1.95E-36 | 0.30 (T/C) | 31.39 | Intergenic | - | - | Replicated | - | 16:38479139 |
| X:96131734 | C14:0 | F_2_ | *-* | 8.49E-09 | 0.41 (T/A) | 6.91 | Intergenic | - | - | Novel | - | X:73409838 |
| X:103467488 | FattyAI | F_2_ | *-* | 1.63E-10 | 0.32 (A/C) | 8.04 | Intergenic | - | - | Novel | - | X:88942546 |
| 6:71008471 | C16:1n-7/C16:0 | Laiwu | *-* | 2.96E-08 | 0.45 (G/T) | 5.61 | Intergenic | - | - | Replicated | - | 6:76754514 |
| 7:27337970 | C18:1n-9/C16:1n-7 | Laiwu | *APOM* | 4.15E-09 | 0.35 (C/T) | 9.13 | Intergenic | - | - | Novel | - | 7:23495730 |
| 7:36084624 | C16:1n-7 | Laiwu | *-* | 2.51E-09 | 0.33 (C/T) | 10.11 | Upstream | Liver_H3K27Ac | - | Enhanced | 2 | 7:31352579 |
| 7:58228023 | C20:2n-6/C18:2n-6 | Laiwu | *-* | 1.52E-10 | 0.35 (C/A) | 19.97 | Intergenic | - | - | Replicated | - | - |
| 7:134418662 | C20:1n-9 | Laiwu | *ELOVL5* | 1.47E-17 | 0.35 (G/T) | 33.75 | Intron | - | - | Replicated | - | 7:46674650 |
| 7:134418662 | C20:1n-9/C18:1n-9 | Laiwu | *ELOVL5* | 4.22E-18 | 0.35 (G/T) | 34.31 | Intron | - | - | Replicated | - | 7:46674650 |
| 7:134418662 | C20:1n-9/C20:0 | Laiwu | *ELOVL5* | 4.76E-17 | 0.49 (T/C) | 37.41 | Intron | - | - | Replicated | - | 7:46674650 |
| 9:13950534 | C16:0/C14:0 | Laiwu | *THRSP* | 2.91E-08 | 0.10 (T/C) | 17.00 | Intron | - | - | Novel | - | 9:12680239 |
| 12:273754 | C14:0 | Laiwu | *FASN* | 4.42E-11 | 0.39 (T/A) | 10.07 | Upstream | Liver_H3K27Ac | - | Replicated | - | 12:464517 |
| 12:1888848 | C16:0/C14:0 | Laiwu | *FASN* | 4.27E-11 | 0.44 (A/C) | 9.34 | Intron | - | - | Novel | - | 12:1987781 |
| 12:57923280 | C20:4n-6/C18:2n-6 | Laiwu | *-* | 1.20E-08 | 0.40 (G/A) | 14.57 | Intron | - | - | Replicated | - | 12:55092844 |
| 12:57937025 | C20:4n-6/C20:3n-6 | Laiwu | *-* | 1.99E-10 | 0.25 (T/C) | 19.34 | Upstream | - | - | Replicated | - | 12:55106595 |
| 12:57973906 | C20:4n-6/C20:2n-6 | Laiwu | *-* | 7.35E-10 | 0.29 (C/G) | 17.49 | Intron | - | - | Replicated | - | 12:55236380 |
| 12:59707351 | FattyTI | Laiwu | *-* | 2.22E-09 | 0.33 (T/C) | 9.64 | Intergenic | Liver_H3K27Ac | - | Enhanced | 3 | 12:56727637 |
| 12:59707351 | SFA | Laiwu | *-* | 1.45E-10 | 0.33 (T/C) | 11.23 | Intergenic | Liver_H3K27Ac | - | Enhanced | 3 | 12:56727637 |
| 15:146109641 | C20:0 | Laiwu | *-* | 3.26E-09 | 0.42 (C/T) | 10.01 | Intergenic | - | - | Novel | - | 15:132335105 |
| 16:36821647 | C20:0/C18:0 | Laiwu | *-* | 1.80E-22 | 0.09 (T/G) | 35.98 | Upstream | - | - | Novel | - | 16:34705907 |
| 16:41971184 | C20:0 | Laiwu | *ELOVL7* | 2.46E-19 | 0.06 (C/T) | 27.81 | Intergenic | - | - | Enhanced | 4 | 16:39105827 |
| 4:63953113 | C18:0 | Sutai | *-* | 9.76E-09 | 0.09 (G/A) | 5.68 | Downstream | - | - | Replicated | - | 4:111590138 |
| 5:73950290 | C20:0/C18:0 | Sutai | *ABCD2* | 2.24E-11 | 0.25 (A/G) | 10.75 | Intron | - | - | Novel | - | 5:71330651 |
| 6:67864540 | C20:3n-6 | Sutai | *-* | 2.52E-08 | 0.27 (T/G) | 7.54 | Intergenic | - | - | Replicated | - | 6:73569713 |
| 13:24928872 | C20:1n-9/C20:0 | Sutai | *ACAA1* | 1.14E-09 | 0.16 (C/T) | 15.95 | Intron | - | - | Novel | - | - |
| 13:165434306 | C20:1n-9 | Sutai | *-* | 3.62E-08 | 0.07 (A/G) | 11.79 | Intergenic | - | - | Novel | - | 13:156117042 |
| 14:120527702 | MUFA | Sutai | *SCD* | 4.32E-08 | 0.35 (T/C) | 10.31 | Intron | - | - | Enhanced | 2 | 14:111081328 |
| 14:121417035 | C18:1n-9/C18:0 | Sutai | *SCD* | 1.16E-12 | 0.36 (G/A) | 19.87 | Intergenic | - | - | Replicated | - | 14:111757382 |
| 14:121469254 | C18:0 | Sutai | *SCD* | 4.71E-15 | 0.28 (A/G) | 19.27 | Intergenic | - | - | Replicated | - | 14:111811103 |
| 14:121469254 | C18:0/C16:0 | Sutai | *SCD* | 2.87E-13 | 0.28 (A/G) | 17.40 | Intergenic | - | - | Replicated | - | 14:111811103 |
| 14:121583641 | SFA | Sutai | *SCD* | 1.60E-08 | 0.12 (A/G) | 13.00 | Intergenic | - | - | Replicated | - | 14:111923271 |
| 16:34617582 | C20:1n-9/C20:0 | Sutai | *-* | 2.45E-08 | 0.30 (G/A) | 16.65 | Intergenic | - | - | Novel | - | 16:32644325 |
| 16:41306957 | C20:0/C18:0 | Sutai | *ELOVL7* | 4.13E-24 | 0.40 (G/A) | 38.41 | Intergenic | - | - | Replicated | - | 16:38609693 |
| 16:42724852 | C20:0 | Sutai | *ELOVL7* | 3.86E-19 | 0.39 (T/C) | 34.78 | Intergenic | - | - | Replicated | - | 16:39798678 |

**Supplementary Table S4**. Summary of significant loci (*p*-value < 5 × 10^-8^) identified for fatty acid composition and metabolism traits by GWAS meta-analysis.

| **chromosome:** | **Trait** | **Candidate** | ***p*-value** | **Weight** | **Zscore** | **Minor/major** | **Variant** | **Overlap** | **Overlap** | **Category** | **Increased** | **Positions of lead** |
| --- | --- | --- | --- | --- | --- | --- | --- | --- | --- | --- | --- | --- |
| **position** |  | **Genes** |  |  |  |  | **Annotation** | **with enhancer** | **with promoter** |  | **significance (units)** | **SNPs on *Sscrofa* 11.1** |
| 2:6999506 | C16:0 | *NAA40* | 2.88E-08 | 925 | 5.549 | A/G | Intron | - | - | Novel | - | 2:7778141 |
| 2:9133024 | C20:3n-6 | *FADS2* | 4.55E-11 | 646 | 6.585 | T/C | Intron | Liver_H3K27Ac | - | Replicated | - | 2:9653127 |
| 2:9133024 | C20:3n-6/C18:2n-6 | *FADS2* | 1.79E-25 | 646 | 10.431 | T/C | Intron | Liver_H3K27Ac | - | Enhanced | 2 | 2:9653127 |
| 2:9134074 | C20:4n-6/C20:3n-6 | *FADS2* | 3.36E-14 | 646 | -7.584 | A/G | Intron | Liver_H3K27Ac | - | Replicated | - | 2:9652077 |
| 2:62618497 | C20:1n-9/C20:0 | *-* | 1.86E-08 | 932 | 5.624 | T/C | Intergenic | - | - | Novel | - | - |
| 2:115831690 | C20:2n-6 | *-* | 5.22E-09 | 610 | -5.84 | A/C | Intergenic | - | - | Replicated | - | 2:111472105 |
| 2:136670441 | C20:2n-6/C18:2n-6 | *SLC27A6* | 3.58E-08 | 627 | 5.51 | T/C | Intergenic | - | - | Novel | - | 2:131395848 |
| 3:58790600 | C18:0 | *-* | 1.46E-08 | 646 | 5.667 | A/T | Intergenic | - | - | Novel | - | 3:56254328 |
| 3:58790600 | C18:1n-9/C18:0 | *ANKRD23* | 3.94E-08 | 646 | -5.494 | A/T | Intergenic | - | - | Novel | - | 3:56254328 |
| 3:91991032 | C20:1n-9 | *-* | 1.15E-08 | 1256 | -5.708 | A/G | Downstream | - | - | Replicated | - | 3:42332979 |
| 3:91991482 | C20:2n-6/C18:2n-6 | *-* | 2.22E-08 | 646 | 5.594 | A/G | Downstream | - | - | Replicated | - | 3:42333429 |
| 3:92060319 | C20:1n-9/C18:1n-9 | *-* | 1.50E-09 | 942 | 6.044 | A/G | Upstream | - | - | Replicated | - | 3:42402016 |
| 3:92116045 | ACL | *-* | 9.63E-09 | 636 | -5.737 | A/C | Intergenic | - | - | Replicated | - | 3:42457740 |
| 4:25204956 | ACL | *-* | 4.64E-08 | 1218 | -5.465 | T/G | Intergenic | - | - | Novel | - | 4:23371727 |
| 4:63923829 | C16:1n-7/C16:0 | *-* | 3.87E-10 | 1497 | -6.259 | A/G | Intron | - | - | Replicated | - | 4:111619429 |
| 4:63923829 | C18:0 | *-* | 1.36E-20 | 1497 | 9.303 | A/G | Intron | - | - | Replicated | - | 4:111619429 |
| 4:63923829 | C18:1n-9/C18:0 | *-* | 5.69E-19 | 1497 | -8.898 | A/G | Intron | - | - | Replicated | - | 4:111619429 |
| 4:63923829 | FattyTI | *-* | 1.95E-12 | 1201 | 7.038 | A/G | Intron | - | - | Replicated | - | 4:111619429 |
| 4:63923829 | MUFA | *-* | 2.79E-08 | 1497 | -5.554 | A/G | Intron | - | - | Replicated | - | 4:111619429 |
| 4:63923829 | MUFA/SFA | *-* | 4.27E-16 | 1497 | -8.131 | A/G | Intron | - | - | Replicated | - | 4:111619429 |
| 4:63923829 | SFA | *-* | 8.04E-14 | 1497 | 7.47 | A/G | Intron | - | - | Replicated | - | 4:111619429 |
| 4:63931931 | C18:0/C16:0 | *-* | 5.57E-13 | 1828 | -7.211 | A/G | Intron | - | - | Replicated | - | 4:111611321 |
| 4:70369475 | C18:2n-6 | *-* | 1.09E-08 | 887 | 5.717 | T/C | Intron | - | - | Novel | - | 4:64738993 |
| 4:70369475 | n-6 | *-* | 5.18E-09 | 887 | 5.841 | T/C | Intron | - | - | Novel | - | 4:64738993 |
| 4:70369475 | PUFA | *-* | 1.65E-08 | 887 | 5.645 | T/C | Intron | - | - | Novel | - | 4:64738993 |
| 4:88240476 | C20:4n-6 | *-* | 1.87E-08 | 1532 | 5.624 | A/G | Intergenic | - | - | Novel | - | 4:80657363 |
| 4:134703308 | C14:0 | *ABCD3* | 3.25E-08 | 627 | 5.527 | T/C | Intergenic | Liver_H3K27Ac | - | Novel | - | 4:123057774 |
| 5:20100162 | FattyTI | *-* | 2.79E-09 | 1247 | -5.943 | T/G | Intergenic | - | - | Novel | - | 5:19341341 |
| 5:73720981 | C20:0 | *ABCD2* | 1.06E-09 | 611 | -6.101 | A/T | Intron | - | - | Replicated | - | 5:71160146 |
| 5:73720981 | C20:0/C18:0 | *ABCD2* | 7.85E-14 | 611 | -7.473 | A/T | Intron | - | - | Enhanced | 4 | 5:71160146 |
| 6:536005 | n-3 | *ACSF3* | 5.99E-09 | 1507 | 5.817 | T/C | Intergenic | - | - | Novel | - | 6:383187 |
| 6:539297 | C18:3n-3 | *ACSF3* | 7.97E-09 | 1211 | -5.769 | A/G | Intergenic | - | - | Novel | - | 6:382189 |
| 6:3110205 | C18:1n-9/C18:0 | *-* | 9.65E-09 | 906 | -5.737 | T/C | Intergenic | - | - | Novel | - | - |
| 6:31165606 | C18:0 | *-* | 1.58E-08 | 1237 | -5.653 | A/G | Intergenic | - | - | Novel | - | 6:35980456 |
| 6:62668102 | C16:1n-7/C16:0 | *ACOT7* | 3.42E-08 | 941 | 5.518 | T/C | Intergenic | - | - | Novel | - | 6:67955949 |
| 6:70177898 | C16:1n-7 | *-* | 2.61E-09 | 636 | -5.954 | T/C | Intron | - | - | Replicated | - | 6:75834603 |
| 6:70184955 | ACL | *-* | 3.76E-09 | 636 | -5.895 | T/C | Upstream | - | - | Replicated | - | 6:75841642 |
| 6:80441149 | C14:0 | *-* | 7.78E-11 | 942 | -6.505 | A/G | Intergenic | - | - | Novel | - | 6:86511849 |
| 6:80441149 | C16:0/C14:0 | *-* | 4.00E-10 | 627 | 6.254 | A/G | Intergenic | - | - | Novel | - | 6:86511849 |
| 6:135724272 | C20:3n-6 | *-* | 1.98E-09 | 1533 | 5.999 | C/G | Intergenic | Liver_H3K27Ac | Liver_H3K4me3 | Novel | - | 6:147319047 |
| 7:9561943 | PUFA/SFA | *-* | 1.37E-09 | 941 | 6.059 | A/G | Intron | - | - | Novel | - | 7:9159182 |
| 7:30507454 | C16:1n-7 | *-* | 2.63E-09 | 951 | -5.953 | A/G | Intergenic | - | - | Replicated | - | 7:26040314 |
| 7:30877027 | C16:0/C14:0 | *-* | 1.36E-08 | 951 | -5.678 | T/C | Intergenic | - | - | Replicated | - | 7:26412583 |
| 7:30900578 | C20:4n-6 | *-* | 7.63E-10 | 925 | 6.152 | T/C | Intergenic | - | - | Replicated | - | 7:26594185 |
| 7:31229695 | ACL | *-* | 3.11E-08 | 2152 | 5.535 | A/G | Intergenic | - | - | Replicated | - | 7:26787303 |
| 7:31500144 | C14:0 | *-* | 8.00E-09 | 1542 | -5.768 | T/C | Intergenic | Liver_H3K27Ac | - | Replicated | - | 7:27057971 |
| 7:31552838 | C16:0 | *-* | 5.89E-10 | 896 | 6.193 | T/C | Intergenic | - | - | Replicated | - | 7:27060666 |
| 7:32460339 | C18:1n-9/C16:1n-7 | *-* | 8.08E-12 | 951 | 6.837 | T/C | Intergenic | - | - | Replicated | - | - |
| 7:34838746 | C18:1n-9 | *-* | 3.82E-10 | 1211 | 6.261 | A/G | Downstream | - | - | Replicated | - | 7:30179365 |
| 7:34838746 | MUFA | *-* | 4.72E-10 | 1211 | 6.228 | A/G | Downstream | - | - | Replicated | - | 7:30179365 |
| 7:34864828 | C20:2n-6 | *-* | 1.24E-15 | 2152 | 8 | A/G | Intron | - | - | Replicated | - | 7:30204804 |
| 7:34908299 | C18:2n-6 | *-* | 8.00E-10 | 906 | 6.145 | A/G | Intron | - | - | Replicated | - | 7:30248155 |
| 7:34913250 | PUFA | *-* | 5.30E-09 | 1202 | -5.838 | T/G | Intron | - | - | Replicated | - | 7:30253105 |
| 7:34928645 | C18:3n-3 | *-* | 1.06E-20 | 1507 | -9.33 | A/G | Upstream | - | - | Enhanced | 5 | 7:30268499 |
| 7:34928645 | n-3 | *-* | 2.40E-19 | 1507 | -8.993 | A/G | Upstream | - | - | Replicated | - | 7:30268499 |
| 7:35432281 | C20:4n-6/C18:2n-6 | *-* | 5.42E-10 | 906 | -6.206 | A/C | Intergenic | Liver_H3K27Ac | - | Replicated | - | - |
| 7:36084624 | C18:0/C16:0 | *-* | 4.58E-12 | 896 | -6.918 | T/C | Upstream | Liver_H3K27Ac | - | Replicated | - | 7:31352579 |
| 7:36087304 | C18:1n-9/C18:0 | *-* | 1.42E-08 | 1211 | 5.672 | A/G | Intron | Liver_H3K27Ac | - | Replicated | - | 7:31349899 |
| 7:36502191 | C20:4n-6/C20:2n-6 | *-* | 1.17E-09 | 2133 | -6.084 | T/C | Intergenic | Liver_H3K27Ac | - | Replicated | - | 7:31574329 |
| 7:98524351 | MUFA/SFA | *-* | 2.81E-08 | 906 | 5.553 | A/T | Intergenic | Liver_H3K27Ac | - | Novel | - | 7:92213572 |
| 7:134543542 | C20:2n-6/C18:2n-6 | *ELOVL5* | 1.65E-21 | 1837 | -9.525 | T/C | Upstream | - | - | Replicated | - | 7:46784214 |
| 7:134556509 | C20:1n-9 | *ELOVL5* | 1.42E-42 | 1227 | 13.676 | T/C | Intergenic | - | - | Replicated | - | 7:46824994 |
| 7:134556509 | C20:1n-9/C18:1n-9 | *ELOVL5* | 9.62E-46 | 1227 | 14.197 | T/C | Intergenic | - | - | Enhanced | 23 | 7:46824994 |
| 8:118240617 | FattyTI | *ELOVL6* | 2.37E-10 | 627 | -6.335 | T/G | Intergenic | - | - | Replicated | - | 8:110352616 |
| 8:119726275 | C16:1n-7 | *ELOVL6* | 1.32E-17 | 1532 | -8.542 | T/C | Intergenic | - | - | Replicated | - | 8:111428042 |
| 8:119726275 | C16:1n-7/C16:0 | *ELOVL6* | 3.71E-12 | 1532 | -6.948 | T/C | Intergenic | - | - | Replicated | - | 8:111428042 |
| 8:119913595 | C18:1n-9 | *ELOVL6* | 4.94E-10 | 1526 | 6.221 | T/C | Intergenic | - | - | Replicated | - | 8:111710419 |
| 8:119942096 | C18:1n-9/C16:1n-7 | *ELOVL6* | 8.51E-24 | 1516 | 10.058 | A/C | Intergenic | - | - | Enhanced | 6 | 8:111739258 |
| 8:119942141 | C18:0/C16:0 | *ELOVL6* | 1.64E-11 | 1516 | 6.735 | A/C | Intergenic | - | - | Replicated | - | 8:111739303 |
| 8:120637780 | FattyAI | *ELOVL6* | 2.03E-12 | 2448 | -7.032 | A/G | Intergenic | - | - | Replicated | - | 8:112616730 |
| 8:120705559 | C16:0 | *ELOVL6* | 8.28E-12 | 1837 | -6.834 | A/G | Intergenic | - | - | Replicated | - | 8:112561241 |
| 9:11302313 | C20:4n-6/C20:3n-6 | *DGAT2* | 3.13E-08 | 331 | -5.534 | A/G | Intron | - | - | Replicated | - | 9:10212537 |
| 9:11880062 | C16:1n-7 | *DGAT2* | 4.46E-09 | 1837 | 5.866 | T/C | Intergenic | - | - | Replicated | - | 9:10769287 |
| 9:11880062 | C16:1n-7/C16:0 | *DGAT2* | 1.84E-10 | 2133 | 6.374 | T/C | Intergenic | - | - | Replicated | - | 9:10769287 |
| 9:13133646 | C18:0/C16:0 | *THRSP* | 1.13E-09 | 1246 | 6.09 | A/C | Intergenic | - | - | Replicated | - | 9:11981882 |
| 9:13143701 | C14:0 | *THRSP* | 1.02E-11 | 1561 | 6.804 | A/G | Intergenic | - | - | Replicated | - | 9:11991583 |
| 9:13698784 | C16:0/C14:0 | *THRSP* | 6.63E-12 | 1561 | 6.865 | T/C | Intron | - | - | Enhanced | 4 | 9:12359706 |
| 9:14505194 | C18:1n-9/C16:1n-7 | *-* | 8.82E-11 | 1821 | 6.486 | T/C | Intron | - | - | Replicated | - | 9:13224122 |
| 9:16594531 | FattyAI | *-* | 3.55E-08 | 941 | 5.512 | A/G | Intergenic | - | - | Novel | - | 9:14942617 |
| 9:16619148 | C16:0 | *-* | 4.04E-09 | 1561 | 5.882 | T/C | Intergenic | - | - | Novel | - | 9:14978810 |
| 9:41039348 | C18:1n-9/C18:0 | *ACAT1* | 1.92E-08 | 611 | 5.619 | A/G | Downstream | - | - | Novel | - | 9:36853890 |
| 9:51656970 | FattyTI | *ABCG4* | 4.54E-08 | 1507 | -5.468 | T/C | Intergenic | - | - | Novel | - | - |
| 9:115117941 | C20:4n-6 | *-* | 1.32E-08 | 932 | 5.684 | T/C | Intergenic | - | - | Novel | - | 9:104385210 |
| 9:115118474 | C20:4n-6/C18:2n-6 | *-* | 2.27E-08 | 1247 | 5.59 | T/C | Intergenic | - | - | Novel | - | 9:104385743 |
| 9:117994281 | C20:3n-6 | *PRKAR2B* | 3.81E-08 | 1532 | -5.499 | A/C | Intron | Liver_H3K27Ac | - | Novel | - | 9:107239414 |
| 9:148465352 | C18:3n-3 | *-* | 3.91E-08 | 1821 | 5.495 | A/G | Intron | - | - | Novel | - | 9:134932478 |
| 10:57716534 | C20:1n-9/C20:0 | *-* | 2.09E-08 | 906 | 5.604 | C/G | Intergenic | - | - | Novel | - | 10:52061036 |
| 11:15790399 | C16:0 | *-* | 9.42E-09 | 1532 | 5.741 | T/G | Intergenic | Liver_H3K27Ac | - | Novel | - | 11:15486995 |
| 11:15790399 | SFA | *-* | 4.53E-08 | 1201 | 5.469 | T/G | Intergenic | Liver_H3K27Ac | - | Novel | - | 11:15486995 |
| 11:49108312 | C18:3n-3 | *-* | 4.16E-10 | 2117 | -6.248 | A/G | Intergenic | - | - | Novel | - | 11:44649504 |
| 11:49108312 | n-3 | *-* | 4.23E-10 | 2117 | -6.245 | A/G | Intergenic | - | - | Novel | - | 11:44649504 |
| 11:76164380 | C18:1n-9 | *PCCA* | 6.56E-09 | 941 | 5.802 | A/G | Intergenic | - | - | Novel | - | 11:68905324 |
| 12:100661 | C20:0 | *FASN* | 1.33E-08 | 636 | 5.682 | A/G | Intron | - | - | Replicated | - | 12:637028 |
| 12:387430 | C20:1n-9 | *FASN* | 3.98E-12 | 331 | 6.938 | T/C | Intron | - | - | Replicated | - | 12:350246 |
| 12:705440 | C20:2n-6/C18:2n-6 | *FASN* | 8.68E-13 | 646 | 7.15 | T/C | Intergenic | - | - | Replicated | - | 12:113587 |
| 12:1176481 | C16:0 | *FASN* | 1.01E-12 | 331 | -7.129 | T/C | Intergenic | - | - | Replicated | - | 12:1333661 |
| 12:1176481 | C16:1n-7 | *FASN* | 8.67E-20 | 331 | -9.104 | T/C | Intergenic | - | - | Replicated | - | 12:1333661 |
| 12:1176481 | C16:1n-7/C16:0 | *FASN* | 1.23E-16 | 331 | -8.281 | T/C | Intergenic | - | - | Replicated | - | 12:1333661 |
| 12:1176481 | C18:1n-9/C16:1n-7 | *FASN* | 2.38E-30 | 331 | 11.449 | T/C | Intergenic | - | - | Replicated | - | 12:1333661 |
| 12:1380908 | C14:0 | *FASN* | 8.12E-21 | 331 | 9.358 | T/G | Intron | - | - | Replicated | - | - |
| 12:1380908 | C16:0/C14:0 | *FASN* | 8.66E-21 | 331 | -9.351 | T/G | Intron | - | - | Replicated | - | - |
| 12:1482194 | C18:1n-9 | *FASN* | 1.32E-11 | 1218 | -6.767 | A/G | Intron | - | - | Replicated | - | 12:1576644 |
| 12:1482194 | MUFA | *FASN* | 2.39E-09 | 1218 | -5.969 | A/G | Intron | - | - | Replicated | - | 12:1576644 |
| 12:1482194 | MUFA/SFA | *FASN* | 5.10E-09 | 922 | -5.844 | A/G | Intron | - | - | Replicated | - | 12:1576644 |
| 12:1485619 | FattyAI | *FASN* | 3.58E-15 | 331 | 7.869 | T/C | Intron | - | - | Replicated | - | 12:1580069 |
| 12:1565371 | DBI | *FASN* | 1.12E-10 | 331 | -6.449 | C/G | Intron | Liver_H3K27Ac | - | Replicated | - | 12:1659672 |
| 12:1565371 | UI | *FASN* | 1.12E-10 | 331 | -6.449 | C/G | Intron | Liver_H3K27Ac | - | Replicated | - | 12:1659672 |
| 12:1569409 | ACL | *FASN* | 9.47E-13 | 331 | 7.138 | A/G | Intron | Liver_H3K27Ac | - | Replicated | - | 12:1663708 |
| 12:1569409 | C18:0/C16:0 | *FASN* | 1.08E-15 | 331 | 8.017 | A/G | Intron | Liver_H3K27Ac | - | Replicated | - | 12:1663708 |
| 12:1569490 | C20:2n-6 | *FASN* | 1.18E-08 | 331 | 5.703 | A/G | Intron | Liver_H3K27Ac | - | Replicated | - | 12:1663789 |
| 12:57919672 | C20:4n-6/C18:2n-6 | *-* | 2.24E-08 | 305 | -5.593 | A/G | Intron | - | - | Replicated | - | 12:55089236 |
| 12:58054827 | C20:4n-6/C20:3n-6 | *-* | 9.53E-09 | 305 | -5.739 | A/T | Intron | - | - | Replicated | - | 12:55422528 |
| 12:59691313 | FattyTI | *-* | 1.13E-08 | 305 | 5.709 | A/C | Intergenic | - | - | Replicated | - | 12:109285649 |
| 12:59691313 | SFA | *-* | 1.01E-09 | 305 | 6.108 | A/C | Intergenic | - | - | Replicated | - | 12:109285649 |
| 12:60294897 | C20:4n-6/C20:2n-6 | *-* | 3.60E-10 | 1837 | 6.27 | T/C | Intergenic | - | - | Replicated | - | 12:57379273 |
| 12:60789959 | C18:0 | *-* | 2.06E-10 | 922 | 6.357 | A/G | Intergenic | - | - | Replicated | - | 12:57870668 |
| 12:60789959 | C18:1n-9/C18:0 | *-* | 5.94E-10 | 922 | -6.192 | A/G | Intergenic | - | - | Replicated | - | 12:57870668 |
| 13:40365857 | ACL | *ACOX2* | 4.00E-09 | 315 | 5.884 | T/G | Intergenic | - | - | Replicated | - | 13:37034334 |
| 13:78511082 | MUFA/SFA | *ACAD9* | 4.30E-08 | 601 | -5.478 | T/C | Intergenic | - | - | Novel | - | 13:70814262 |
| 13:91132399 | C16:1n-7/C16:0 | *-* | 3.84E-09 | 1561 | -5.891 | A/G | Intergenic | - | - | Novel | - | 13:83336976 |
| 13:93981361 | C18:1n-9/C16:1n-7 | *-* | 8.39E-09 | 1230 | -5.761 | A/G | Intergenic | - | - | Novel | - | 13:86244041 |
| 13:165447273 | C20:1n-9 | *-* | 5.54E-10 | 601 | -6.203 | T/C | Intergenic | - | - | Replicated | - | 13:156130001 |
| 13:165447273 | C20:1n-9/C18:1n-9 | *-* | 7.14E-09 | 601 | -5.788 | T/C | Intergenic | - | - | Replicated | - | 13:156130001 |
| 13:216170783 | C16:1n-7 | *ABCG1* | 2.99E-08 | 636 | -5.542 | A/G | Intron | - | - | Novel | - | 13:205766608 |
| 14:121398370 | C18:0 | *SCD* | 7.34E-50 | 1812 | -14.846 | T/C | Intergenic | - | - | Enhanced | 17 | 14:111738759 |
| 14:121398370 | C18:0/C16:0 | *SCD* | 1.82E-36 | 1812 | -12.612 | T/C | Intergenic | - | - | Replicated | - | 14:111738759 |
| 14:121410631 | C18:1n-9/C18:0 | *SCD* | 1.53E-44 | 1812 | -14.001 | T/G | Intergenic | - | - | Replicated | - | 14:111750998 |
| 14:121412476 | C16:0 | *SCD* | 4.15E-10 | 1542 | 6.248 | T/C | Intergenic | - | - | Replicated | - | 14:111752840 |
| 14:121417185 | UI | *SCD* | 6.62E-14 | 1221 | -7.495 | A/G | Intergenic | - | - | Replicated | - | 14:111757531 |
| 14:121418492 | FattyTI | *SCD* | 1.25E-23 | 1821 | 10.02 | T/G | Intergenic | - | - | Replicated | - | 14:111758839 |
| 14:121425536 | FattyAI | *SCD* | 8.54E-15 | 906 | 7.759 | A/G | Intergenic | - | - | Replicated | - | 14:111765859 |
| 14:121452077 | C20:1n-9/C20:0 | *SCD* | 9.58E-10 | 2133 | -6.116 | A/G | Intergenic | - | - | Replicated | - | 14:111792489 |
| 14:121457984 | DBI | *SCD* | 6.09E-14 | 1221 | -7.506 | A/C | Intergenic | - | - | Replicated | - | 14:111799788 |
| 14:121458036 | SFA | *SCD* | 6.29E-30 | 2117 | 11.364 | A/G | Intergenic | - | - | Replicated | - | 14:111799839 |
| 14:121472273 | MUFA/SFA | *SCD* | 3.95E-32 | 1802 | 11.799 | A/G | Intergenic | - | - | Replicated | - | 14:111814110 |
| 14:121545231 | C16:1n-7 | *SCD* | 9.30E-20 | 1828 | -9.097 | T/C | Intron | - | - | Replicated | - | 14:111887022 |
| 14:121545231 | C16:1n-7/C16:0 | *SCD* | 1.40E-25 | 1828 | -10.454 | T/C | Intron | - | - | Replicated | - | 14:111887022 |
| 14:121545231 | C18:1n-9/C16:1n-7 | *SCD* | 7.17E-14 | 1828 | 7.485 | T/C | Intron | - | - | Replicated | - | 14:111887022 |
| 14:121545583 | C18:1n-9 | *SCD* | 1.27E-12 | 1497 | 7.098 | A/G | Intron | - | - | Replicated | - | 14:111887374 |
| 14:121545583 | MUFA | *SCD* | 3.01E-19 | 1497 | 8.968 | A/G | Intron | - | - | Replicated | - | 14:111887374 |
| 15:146109641 | C20:0 | *-* | 3.26E-09 | 305 | -5.918 | T/C | Intergenic | - | - | Replicated | - | 15:132335105 |
| 16:43507850 | C20:0 | *ELOVL7* | 7.29E-71 | 1802 | -17.798 | T/C | Intergenic | - | - | Replicated | - | 16:40507815 |
| 16:43507850 | C20:0/C18:0 | *ELOVL7* | 3.80E-95 | 1802 | -20.696 | T/C | Intergenic | - | - | Enhanced | 32 | 16:40507815 |
| 16:43507850 | C20:1n-9/C20:0 | *ELOVL7* | 3.23E-32 | 1802 | 11.816 | T/C | Intergenic | - | - | Replicated | - | 16:40507815 |
| 16:45042522 | C20:1n-9 | *-* | 1.02E-10 | 1802 | -6.465 | T/C | Intergenic | - | - | Novel | - | 16:42024402 |
| 16:45042522 | C20:1n-9/C18:1n-9 | *-* | 5.70E-11 | 1802 | -6.551 | T/C | Intergenic | - | - | Novel | - | 16:42024402 |
| 16:57806916 | PUFA/SFA | *-* | 3.80E-08 | 942 | -5.5 | T/C | Intergenic | - | - | Novel | - | 16:53465536 |
| 16:79672852 | C16:0 | *-* | 6.33E-09 | 646 | 5.808 | T/C | Intergenic | - | - | Novel | - | 16:73667630 |
| 17:58003115 | C20:4n-6/C20:3n-6 | *PTGIS* | 3.45E-09 | 1202 | -5.909 | T/G | Intergenic | - | - | Novel | - | 17:51779488 |
| 18:9785748 | C20:3n-6 | *-* | 4.80E-09 | 1221 | 5.854 | T/C | Synonymous | - | - | Novel | - | 18:9280255 |
| X:63518603 | FattyAI | *AWAT1, AWAT2* | 1.01E-10 | 1202 | 6.466 | T/G | Intron | - | - | Novel | - | - |
| X:67734281 | SFA | *-* | 6.68E-11 | 1533 | -6.528 | A/G | Intergenic | - | - | Novel | - | X:59813860 |
| X:73586810 | C16:0 | *-* | 2.03E-10 | 1533 | -6.359 | A/T | Intergenic | - | - | Novel | - | X:63880646 |
| X:96131734 | C14:0 | *-* | 8.49E-09 | 591 | 5.759 | A/T | Intergenic | - | - | Replicated | - | X:73409838 |

**Supplementary Table S5**. Gene ontology and KEGG pathway enrichment analysis of the 32 most plausible candidate genes detected by GWAS and GWAS meta-analysis.

| **GOID** | **GOTerm** | **Number** | ***p*-value** | **Adjusted** | **Associated Genes Found** |
| --- | --- | --- | --- | --- | --- |
|  |  | **of genes** |  | ***p*-value** |  |
| GO:0000038 | very long-chain fatty acid metabolic process | 7 | 2.71E-13 | 9.84E-13 | *ABCD2, ABCD3, ACAA1, ACSBG1, ELOVL5, ELOVL7, SLC27A6* |
| KEGG:00062 | Fatty acid elongation | 5 | 1.99E-09 | 4.30E-09 | *ACAA2, ACOT7, ELOVL5, ELOVL6, ELOVL7* |
| KEGG:00071 | Fatty acid degradation | 4 | 2.51E-06 | 3.68E-06 | *ACAA1, ACAA2, ACAT1, ACSBG1* |
| KEGG:00280 | Valine, leucine and isoleucine degradation | 5 | 6.19E-08 | 1.12E-07 | *ACAA1, ACAA2, ACAT1, ACSF3, PCCA* |
| KEGG:01040 | Biosynthesis of unsaturated fatty acids | 7 | 3.30E-14 | 1.34E-13 | *ACAA1, ACOT7, ELOVL5, ELOVL6, FADS1, FADS2, SCD* |
| GO:0001676 | long-chain fatty acid metabolic process | 12 | 3.30E-18 | 7.60E-17 | *ACAA1, ACOT7, ACSBG1, AWAT1, ELOVL5, ELOVL6,* |
|  |  |  |  |  | *FADS1, FADS2, PTGIS, SLC27A6* |
| KEGG:02010 | ABC transporters | 4 | 2.51E-06 | 3.68E-06 | *ABCD2, ABCD3, ABCG1, ABCG4* |
| KEGG:03320 | PPAR signaling pathway | 6 | 8.04E-09 | 1.63E-08 | *ACAA1, ACOX2, ACSBG1, FADS2, SCD, SLC27A6* |
| GO:0003988 | acetyl-CoA C-acyltransferase activity | 3 | 5.40E-07 | 8.87E-07 | *ACAA1, ACAA2, ACAT1* |
| KEGG:04146 | Peroxisome | 4 | 3.19E-05 | 3.79E-05 | *ABCD2, ABCD3, ACAA1, ACOX2* |
| GO:0005319 | lipid transporter activity | 5 | 4.57E-06 | 6.44E-06 | *ABCD3, ABCG1, ABCG4, APOM, MTTP* |
| GO:0006631 | fatty acid metabolic process | 26 | 3.18E-34 | 2.19E-32 | *ABCD2, ABCD3, ACAA1, ACAA2, ACAD9, ACOT7, ACOX2,* |
|  |  |  |  |  | *ACSBG1, ACSF3, AWAT1, DGAT2, ELOVL5, ELOVL6, ELOVL7, FADS1,* |
|  |  |  |  |  | *FADS2, FASN, PCCA, PLIN5, PRKAR2B, PTGIS, SCD, SLC27A6, SNCA* |
| GO:0006633 | fatty acid biosynthetic process | 13 | 1.15E-17 | 1.99E-16 | *ABCD3, ACOT7, ACSF3, ELOVL5, ELOVL6, ELOVL7, FADS1, FADS2, FASN, PTGIS, SCD* |
| GO:0006635 | fatty acid beta-oxidation | 7 | 2.46E-10 | 5.66E-10 | *ABCD2, ABCD3, ACAA1, ACAD9, ACOX2, PCCA, PLIN5* |
| GO:0006636 | unsaturated fatty acid biosynthetic process | 7 | 2.23E-10 | 5.32E-10 | *ELOVL5, FADS1, FADS2, PTGIS, SCD* |
| GO:0006637 | acyl-CoA metabolic process | 10 | 5.91E-15 | 3.14E-14 | *ACOT7, ACSBG1, ACSF3, DGAT2, ELOVL5, ELOVL6, ELOVL7, FASN, SCD, SNCA* |
| GO:0006638 | neutral lipid metabolic process | 14 | 4.68E-19 | 1.62E-17 | *ACOT7, ACSBG1, ACSF3, AWAT1, DGAT2, ELOVL5,* |
|  |  |  |  |  | *ELOVL6, ELOVL7, FASN, MTTP, PLIN5, SCD, SNCA, THRSP* |
| GO:0006639 | acylglycerol metabolic process | 13 | 2.56E-17 | 3.53E-16 | *ACOT7, ACSBG1, ACSF3, AWAT1, DGAT2, ELOVL5,* |
|  |  |  |  |  | *ELOVL6, ELOVL7, FASN, MTTP, PLIN5, SCD, THRSP* |
| GO:0006641 | triglyceride metabolic process | 12 | 7.01E-16 | 4.84E-15 | *ACOT7, ACSBG1, ACSF3, DGAT2, ELOVL5, ELOVL6,* |
|  |  |  |  |  | *ELOVL7, FASN, MTTP, PLIN5, SCD, THRSP* |
| GO:0006690 | icosanoid metabolic process | 5 | 4.19E-06 | 6.02E-06 | *AWAT1, FADS1, PTGIS* |
| GO:0008374 | O-acyltransferase activity | 3 | 1.13E-04 | 1.24E-04 | *AWAT1, AWAT2, DGAT2* |
| GO:0009062 | fatty acid catabolic process | 8 | 2.43E-11 | 6.70E-11 | *ABCD2, ABCD3, ACAA1, ACAD9, ACOT7, ACOX2, PCCA, PLIN5* |
| GO:0009108 | coenzyme biosynthetic process | 9 | 1.56E-11 | 4.69E-11 | *ACOT7, ACSBG1, ACSF3, ELOVL5, ELOVL6, ELOVL7, FASN, SCD, SNCA* |
| GO:0010866 | regulation of triglyceride biosynthetic process | 3 | 9.18E-06 | 1.22E-05 | *DGAT2, PLIN5, THRSP* |
| GO:0015918 | sterol transport | 4 | 2.50E-05 | 3.07E-05 | *ABCG1, ABCG4, APOM, LIPG* |
| GO:0016053 | organic acid biosynthetic process | 14 | 5.79E-15 | 3.33E-14 | *ABCD3, ACOT7, ACOX2, ACSF3, ELOVL5, ELOVL6,* |
|  |  |  |  |  | *ELOVL7, FADS1, FADS2, FASN, PTGIS, SCD* |
| GO:0016408 | C-acyltransferase activity | 3 | 9.18E-06 | 1.22E-05 | *ACAA1, ACAA2, ACAT1* |
| GO:0016627 | oxidoreductase activity, acting on the CH-CH group of donors | 4 | 7.14E-06 | 9.66E-06 | *ACAA1, ACAD9, ACOX2, FASN* |
| GO:0016717 | oxidoreductase activity, acting on paired donors, with oxidation of a pair of donors | 3 | 9.68E-08 | 1.71E-07 | *FADS1, FADS2, SCD* |
|  | resulting in the reduction of molecular oxygen to two molecules of water |  |  |  |  |
| GO:0016747 | transferase activity, transferring acyl groups other than amino-acyl groups | 10 | 4.58E-11 | 1.21E-10 | *ACAA1, ACAA2, ACAT1, AWAT1, AWAT2, DGAT2, ELOVL5, ELOVL6, FASN, NAA40* |
| GO:0016877 | ligase activity, forming carbon-sulfur bonds | 3 | 8.40E-05 | 9.35E-05 | *ACSBG1, ACSF3, SLC27A6* |
| GO:0019369 | arachidonic acid metabolic process | 5 | 3.14E-07 | 5.28E-07 | *AWAT1, FADS1, PTGIS* |
| GO:0019395 | fatty acid oxidation | 9 | 9.49E-13 | 3.27E-12 | *ABCD2, ABCD3, ACAA1, ACAD9, ACOX2, DGAT2, PCCA, PLIN5* |
| GO:0019432 | triglyceride biosynthetic process | 11 | 3.48E-17 | 3.43E-16 | *ACOT7, ACSBG1, ACSF3, DGAT2, ELOVL5,* |
|  |  |  |  |  | *ELOVL6, ELOVL7, FASN, PLIN5, SCD, THRSP* |
| GO:0019915 | lipid storage | 3 | 3.86E-04 | 3.98E-04 | *ABCG1, DGAT2, PLIN5* |
| GO:0030258 | lipid modification | 10 | 1.85E-11 | 5.31E-11 | *ABCD2, ABCD3, ABCG1, ACAA1, ACAD9, ACOX2, DGAT2, PCCA, PLIN5* |
| GO:0030301 | cholesterol transport | 4 | 2.25E-05 | 2.82E-05 | *ABCG1, ABCG4, APOM, LIPG* |
| GO:0030497 | fatty acid elongation | 3 | 2.11E-06 | 3.23E-06 | *ELOVL5, ELOVL6, ELOVL7* |
| GO:0033293 | monocarboxylic acid binding | 3 | 5.88E-04 | 5.88E-04 | *ACOX2, PCCA, SNCA* |
| GO:0033344 | cholesterol efflux | 3 | 1.29E-04 | 1.39E-04 | *ABCG1, ABCG4, APOM* |
| GO:0033559 | unsaturated fatty acid metabolic process | 9 | 6.94E-12 | 2.18E-11 | *ACAA1, AWAT1, ELOVL5, FADS1, FADS2, PTGIS, SCD* |
| GO:0034367 | macromolecular complex remodeling | 3 | 2.74E-05 | 3.31E-05 | *ABCG1, APOM, LIPG* |
| GO:0034368 | protein-lipid complex remodeling | 3 | 2.74E-05 | 3.31E-05 | *ABCG1, APOM, LIPG* |
| GO:0034369 | plasma lipoprotein particle remodeling | 3 | 2.74E-05 | 3.31E-05 | *ABCG1, APOM, LIPG* |
| GO:0034375 | high-density lipoprotein particle remodeling | 3 | 5.33E-06 | 7.36E-06 | *ABCG1, APOM, LIPG* |
| GO:0034381 | plasma lipoprotein particle clearance | 3 | 5.06E-05 | 5.82E-05 | *APOM, DGAT2, LIPG* |
| GO:0034440 | lipid oxidation | 9 | 1.13E-12 | 3.72E-12 | *ABCD2, ABCD3, ACAA1, ACAD9, ACOX2, DGAT2, PCCA, PLIN5* |
| GO:0035336 | long-chain fatty-acyl-CoA metabolic process | 9 | 3.66E-16 | 2.81E-15 | *ACOT7, ACSBG1, ACSF3, DGAT2, ELOVL5, ELOVL6, ELOVL7, FASN, SCD* |
| GO:0035337 | fatty-acyl-CoA metabolic process | 9 | 1.25E-15 | 7.81E-15 | *ACOT7, ACSBG1, ACSF3, DGAT2, ELOVL5, ELOVL6, ELOVL7, FASN, SCD* |
| GO:0035338 | long-chain fatty-acyl-CoA biosynthetic process | 8 | 2.26E-14 | 1.04E-13 | *ACOT7, ACSBG1, ACSF3, ELOVL5, ELOVL6, ELOVL7, FASN, SCD* |
| GO:0035357 | peroxisome proliferator activated receptor signaling pathway | 3 | 9.18E-06 | 1.22E-05 | *PLIN5, PTGIS* |
| GO:0035358 | regulation of peroxisome proliferator activated receptor signaling pathway | 3 | 2.11E-06 | 3.23E-06 | *PLIN5, PTGIS* |
| GO:0035383 | thioester metabolic process | 10 | 5.91E-15 | 3.14E-14 | *ACOT7, ACSBG1, ACSF3, DGAT2, ELOVL5, ELOVL6, ELOVL7, FASN, SCD, SNCA* |
| GO:0035384 | thioester biosynthetic process | 9 | 1.28E-14 | 6.31E-14 | *ACOT7, ACSBG1, ACSF3, ELOVL5, ELOVL6, ELOVL7, FASN, SCD, SNCA* |
| GO:0036109 | alpha-linolenic acid metabolic process | 4 | 2.64E-08 | 4.92E-08 | *ACAA1, ELOVL5, FADS1, FADS2* |
| GO:0042632 | cholesterol homeostasis | 5 | 7.75E-07 | 1.24E-06 | *ABCG1, APOM, DGAT2, LIPG, MTTP* |
| GO:0042759 | long-chain fatty acid biosynthetic process | 4 | 4.09E-09 | 8.55E-09 | *ACOT7, ELOVL6* |
| GO:0043651 | linoleic acid metabolic process | 4 | 1.94E-08 | 3.71E-08 | *ELOVL5, FADS1, FADS2* |
| GO:0043691 | reverse cholesterol transport | 3 | 1.08E-05 | 1.38E-05 | *ABCG1, APOM, LIPG* |
| GO:0044242 | cellular lipid catabolic process | 9 | 3.66E-10 | 8.15E-10 | *ABCD2, ABCD3, ACAA1, ACAD9, ACOT7, ACOX2, LIPG, PCCA, PLIN5* |
| GO:0045017 | glycerolipid biosynthetic process | 13 | 2.42E-14 | 1.04E-13 | *ACOT7, ACSBG1, ACSF3, AWAT1, DGAT2, ELOVL5,* |
|  |  |  |  |  | *ELOVL6, ELOVL7, FASN, PLIN5, SCD, THRSP* |
| GO:0046320 | regulation of fatty acid oxidation | 3 | 4.18E-05 | 4.89E-05 | *ABCD2, DGAT2, PLIN5* |
| GO:0046394 | carboxylic acid biosynthetic process | 14 | 5.79E-15 | 3.33E-14 | *ABCD3, ACOT7, ACOX2, ACSF3, ELOVL5, ELOVL6,* |
|  |  |  |  |  | *ELOVL7, FADS1, FADS2, FASN, PTGIS, SCD* |
| GO:0046456 | icosanoid biosynthetic process | 4 | 9.38E-06 | 1.22E-05 | *FADS1, PTGIS* |
| GO:0046460 | neutral lipid biosynthetic process | 11 | 4.51E-17 | 3.89E-16 | *ACOT7, ACSBG1, ACSF3, DGAT2, ELOVL5,* |
|  |  |  |  |  | *ELOVL6, ELOVL7, FASN, PLIN5, SCD, THRSP* |
| GO:0046463 | acylglycerol biosynthetic process | 11 | 4.51E-17 | 3.89E-16 | *ACOT7, ACSBG1, ACSF3, DGAT2, ELOVL5,* |
|  |  |  |  |  | *ELOVL6, ELOVL7, FASN, PLIN5, SCD, THRSP* |
| GO:0046889 | positive regulation of lipid biosynthetic process | 3 | 5.65E-04 | 5.73E-04 | *ABCG1, DGAT2, PLIN5* |
| GO:0046949 | fatty-acyl-CoA biosynthetic process | 8 | 3.42E-14 | 1.31E-13 | *ACOT7, ACSBG1, ACSF3, ELOVL5, ELOVL6, ELOVL7, FASN, SCD* |
| GO:0050746 | regulation of lipoprotein metabolic process | 3 | 5.33E-06 | 7.36E-06 | *APOM, DGAT2, LIPG* |
| GO:0051188 | cofactor biosynthetic process | 9 | 8.08E-11 | 2.07E-10 | *ACOT7, ACSBG1, ACSF3, ELOVL5, ELOVL6, ELOVL7, FASN, SCD, SNCA* |
| GO:0055081 | anion homeostasis | 3 | 1.57E-04 | 1.67E-04 | *ABCG1, DGAT2, LIPG* |
| GO:0055088 | lipid homeostasis | 7 | 1.48E-08 | 2.92E-08 | *ABCG1, ACAD9, ACOX2, APOM, DGAT2, LIPG, MTTP* |
| GO:0055092 | sterol homeostasis | 5 | 7.75E-07 | 1.24E-06 | *ABCG1, APOM, DGAT2, LIPG, MTTP* |
| GO:0071616 | acyl-CoA biosynthetic process | 9 | 1.28E-14 | 6.31E-14 | *ACOT7, ACSBG1, ACSF3, ELOVL5, ELOVL6, ELOVL7, FASN, SCD, SNCA* |
| GO:0071825 | protein-lipid complex subunit organization | 4 | 2.28E-06 | 3.42E-06 | *ABCG1, APOM, LIPG, MTTP* |
| GO:0071827 | plasma lipoprotein particle organization | 4 | 1.70E-06 | 2.66E-06 | *ABCG1, APOM, LIPG, MTTP* |
| GO:0072329 | monocarboxylic acid catabolic process | 8 | 1.43E-10 | 3.53E-10 | *ABCD2, ABCD3, ACAA1, ACAD9, ACOT7, ACOX2, PCCA, PLIN5* |
| GO:0072330 | monocarboxylic acid biosynthetic process | 14 | 3.29E-17 | 3.78E-16 | *ABCD3, ACOT7, ACOX2, ACSF3, ELOVL5,* |
|  |  |  |  |  | *ELOVL6, ELOVL7, FADS1, FADS2, FASN, PTGIS, SCD* |
| GO:0090207 | regulation of triglyceride metabolic process | 3 | 6.05E-05 | 6.85E-05 | *DGAT2, PLIN5, THRSP* |
| GO:0097006 | regulation of plasma lipoprotein particle levels | 5 | 2.29E-07 | 3.95E-07 | *ABCG1, APOM, DGAT2, LIPG, MTTP* |
| GO:1901568 | fatty acid derivative metabolic process | 5 | 4.19E-06 | 6.02E-06 | *AWAT1, FADS1, PTGIS* |
| GO:1901570 | fatty acid derivative biosynthetic process | 4 | 9.38E-06 | 1.22E-05 | *FADS1, PTGIS* |
